# Bottom‐Up Programming of Cell States in Cancer Organoids with Defined Synthetic Adhesion Cues

**DOI:** 10.1002/adma.202517390

**Published:** 2026-04-14

**Authors:** Ali Nadernezhad, Verena J. Kast, Dagmar Pette, Franziska Baenke, Daniel E. Stange, Carsten Werner, Daniela Loessner

**Affiliations:** ^1^ Division of Polymer Biomaterials Science Leibniz Institute of Polymer Research Dresden Dresden Germany; ^2^ Department of Visceral Thoracic and Vascular Surgery University Hospital Carl Gustav Carus Medical Faculty Dresden University of Technology Dresden Germany; ^3^ National Center for Tumor Diseases Dresden (NCT/UCC) a partnership between DKFZ Faculty of Medicine and University Hospital Carl Gustav Carus Dresden University of Technology and Helmholtz‐Zentrum Dresden‐Rossendorf (HZDR) Dresden Germany; ^4^ German Cancer Consortium (DKTK), Partner Site Dresden German Cancer Research Center (DKFZ) Dresden Germany; ^5^ Center for Regenerative Therapies Dresden Dresden University of Technology Dresden Germany; ^6^ Department of Chemical and Biological Engineering and Department of Materials Science and Engineering Faculty of Engineering Monash University Melbourne Australia; ^7^ Department of Anatomy and Developmental Biology Biomedicine Discovery Institute Faculty of Medicine Nursing and Health Sciences Monash University Melbourne Australia

**Keywords:** computational biomaterials design, organoid microenvironment engineering, patient‐derived cancer organoids, synthetic extracellular matrix, transcriptomic state programming

## Abstract

Despite advances in defined culture systems, current organoid models lack programmable control of transcriptomic states beyond fixed genetic constraints or broadly specific microenvironmental conditions. Here, a bottom‐up biomaterial‐based platform is introduced to program cell state changes in pancreatic cancer organoids by tuning minimal adhesion cues within a synthetic matrix. A Design of Experiments framework is used to systematically model the patient‐specific transcriptome‐wide impact of matrix‐presented adhesion cues. Focusing on epithelial‐mesenchymal transition (EMT) as a proof‐of‐concept cellular program, a multiobjective optimization approach is applied to identify patient‐specific matrix compositions that enrich EMT‐associated transcriptional programs. Organoids cultured in these optimized matrices exhibit transcriptomic signatures consistent with EMT enrichment and coordinated shift in EMT‐associated regulatory signatures. Secretome profiling further reveals changes in cytokines previously linked to EMT‐associated inflammatory, hypoxia, and TGF‐β signaling. Together, these findings demonstrate that quantitative and targeted modulation of defined adhesion cues enables programmable control of transcriptomic states in pancreatic cancer organoids.

## Graphical abstract

A bottom‐up biomaterial platform is developed to program transcriptomic states in pancreatic cancer organoids by tuning adhesion cues within synthetic matrices. By combining a Design of Experiments framework with multiobjective optimization, matrix compositions are identified that enrich specific cellular programs like EMT. This quantitative approach enables precise control over cell behavior through defined microenvironmental inputs rather than genetic modification.

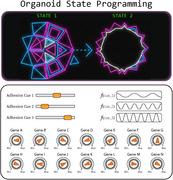

## 1 Introduction

Cancer organoids recapitulate the complex architecture and tumor‐intrinsic tissue heterogeneity, driven by the capacity of cells to self‐organize in response to environmental cues [1]. Harnessing this self‐organization capacity for directed programming of organoid behavior has been pursued through genetic manipulation, including the introduction of oncogenic mutations and inducible gene circuits [1, 2]. However, while genetically engineered organoid models have yielded robust proof‐of‐concept, they remain rigid, require extensive manipulation, and are limited by variable delivery and editing efficiencies across organoid lines. Crucially, these models typically fail to capture the dynamic spatiotemporal complexities of the tumor microenvironment (TME) [1].

Microenvironment‐driven signaling orchestrates cell state transitions and phenotypic plasticity by dynamically regulating inter‐ and intracellular pathways, transcriptional programs, and cellular interactions [3]. This signaling involves complex networks of soluble factors, extracellular matrix (ECM) components, and cell–cell contacts, collectively influencing cellular behavior and directing tissue development, homeostasis, and disease progression. Strategies that leverage these signaling mechanisms allow the dynamic engineering of cellular behavior, positioning them as promising alternatives to genetic engineering.

Engineering the extracellular microenvironment offers a compelling approach by utilizing external cues to guide cell fate. However, achieving precise and predictable control over transcriptional outcomes remains a significant challenge. Current concepts mainly involve increasing TME complexity by incorporating stromal components to initiate tumor‐stroma crosstalk [2, 4, 5] or engineering the ECM to accommodate broader biological functionalities [6, 7]. Despite their promise, these strategies primarily aimed to mimic the general features of TME in diseased tissue rather than establishing a direct causal link between the engineered microenvironment and the specific cancer states. Consequently, these models may fall short in addressing scenarios where target cellular behaviors diverge from general microenvironmental conditions. To overcome these limitations and enable precise control over cancer states, new methodologies must focus on directly coupling microenvironmental cues with defined cell states.

To address this issue, we reasoned that systematic, bottom‐up programming of cancer organoids could be achieved by integrating controlled modulation of cell‐ECM interactions with quantitative gene expression modeling. This approach will enable the definition of microenvironmental conditions that drive global transcriptomic reprogramming to align with specific experimental goals.

## 2 Results

**FIGURE 1:**
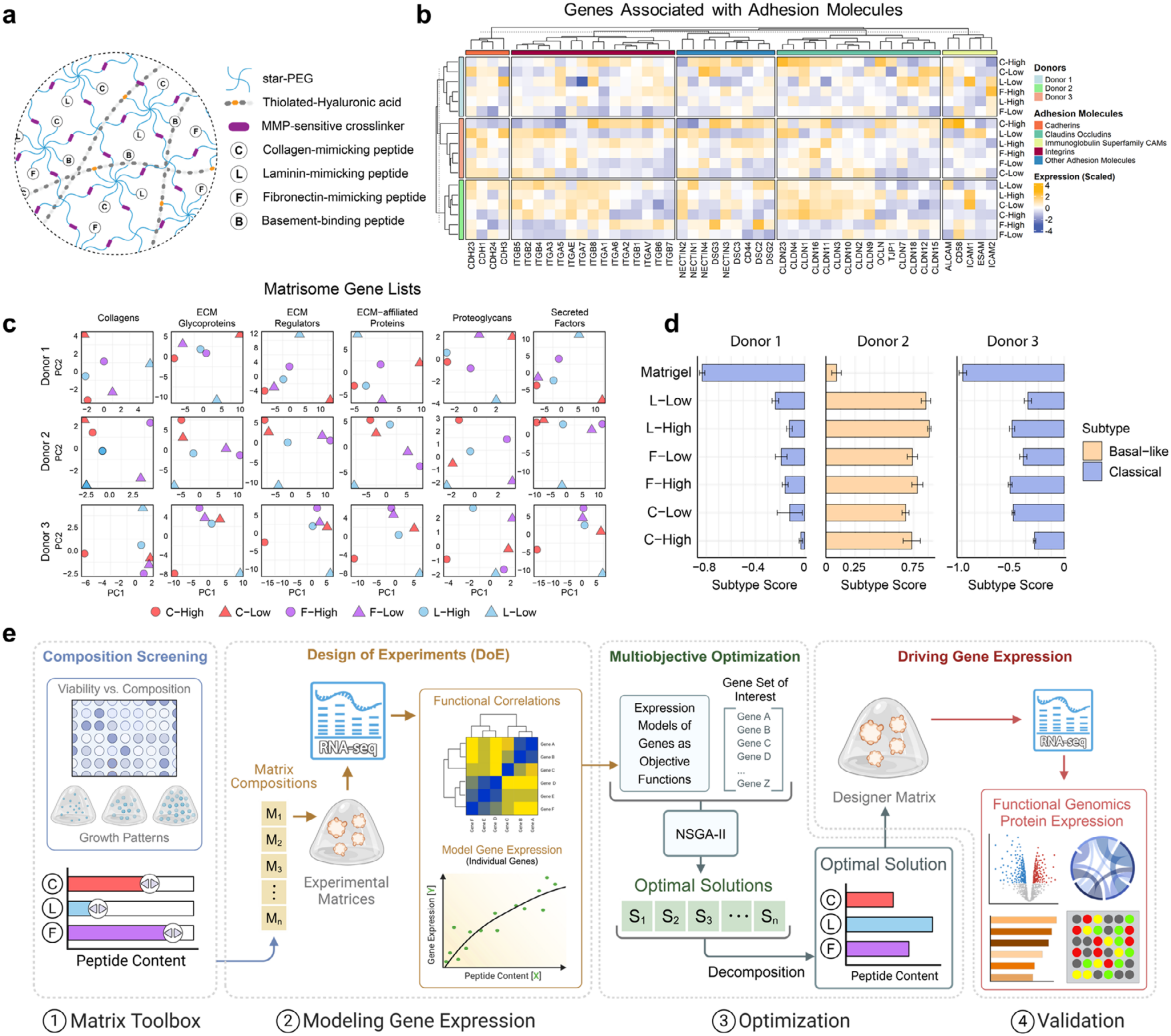
A bottom‐up design to capture and construct transcriptional heterogeneities in pancreatic cancer organoids. (a) Schematic of the modular synthetic matrix system, showing the key biofunctional components and matrix architecture. (b) Heatmap showing donor‐scaled expression patterns of cell adhesion‐related genes in organoids cultured in different matrix compositions. For each peptide‐specific condition (“High” or “Low”), the molar content of the respective peptide was set to the maximum or minimum, respectively, while maintaining the opposite levels for the other peptides. Peptide B content remained constant across all conditions. (c) PCA visualization illustrating variability in expression of core and matrisome‐associated genes across synthetic matrix compositions. (d) Molecular subtype scores derived from RNA‐sequencing, demonstrating a shift toward a more basal‐like phenotype upon culture in synthetic matrices. (e) Schematic workflow outlining the implemented bottom‐up strategy to engineer transcriptional events. (1) A matrix toolbox was identified, and general cell growth and adhesion dynamics in different compositions were evaluated. (2) Gene expression modeling based on the matrix composition was performed using a Design of Experiments (DoE) approach. (3) Gene expression models of selected gene sets were processed using multiobjective optimization algorithms, and the optimal solution, as the matrix composition for a given gene set, was identified. (4) Organoids cultured in the optimal synthetic matrix were sequenced, and the success of the transcriptome reprogramming was evaluated through functional genomics and protein expression analysis. Parts of the schematic were created using BioRender.com.

To build a platform for programming cell states by manipulating cell‐ECM interactions, we used published matrisome data on pancreatic cancer [8, 9, 10] to identify key ECM components of the pancreatic TME, including collagens type I and IV, fibronectin, laminin, and hyaluronic acid. Gene expression analysis of tumor (*n* = 179) and normal pancreas (*n* = 171) tissues from The Cancer Genome Atlas (TCGA) and Genome‐Tissue Expression project (GTEx) confirmed that several of these ECM components were consistently overrepresented in diseased tissues (Figure S1). Based on these findings, we developed our platform using star‐shaped polyethylene glycol (starPEG) hydrogels decorated with short synthetic peptides mimicking the essential pancreatic TME ligands (Figure 1a). Moreover, since pancreatic cancer is characterized by a highly desmoplastic, mechanically stiff TME (5.46 ± 3.18 kPa in cancerous vs 1.06 ± 0.25 kPa in normal tissues) [9 ^,^ 11], impacting mechanotransduction signaling and therapeutic response, we tuned its mechanical properties to match the elevated stiffness of pancreatic tumor tissues (Figure S2).

The synthetic matrices were designed to systematically control cell‐ECM signaling by varying the presentation of three well‐established peptides [7] mimicking collagen type I (**C**), laminin‐1 (**L**), and fibronectin (**F**). A peptide with affinity for cell‐secreted basement membrane proteins, including collagen type IV and laminin [12], was included in the design (**B**). Combining high and low levels of each peptide using molar ratios resulted in distinct expression patterns in cell adhesion‐related genes, suggesting that tuning of cell‐ECM signals elicits transcriptional changes in a controlled environment (Figure 1b). These changes extended beyond cell adhesion molecules as principal component analysis (PCA) revealed differential expression of matrisome‐associated genes [13] in response to both the identity and quantity of the presented peptides (Figure 1c). The transcriptional response to cell adhesion cues varied by patient, as reflected in distinct principal component structures and gene loading patterns for each organoid (Data S1). This result highlights interpatient heterogeneity in shaping matrix‐driven signaling, emphasizing the necessity for patient‐specific approaches in transcriptional programming.

Molecular subtypes of pancreatic cancer correlate with clinical outcomes and responses to therapy and provide a basis for patient stratification and personalized treatments [14, 15]. Beyond their clinical relevance, molecular subtypes reflect the cellular heterogeneity within the pancreatic TME [16]. Recent studies have demonstrated that molecular subtype plasticity in pancreatic cancer organoids is driven by niche‐derived and microenvironmental signals [17, 18, 19]. We observed that the molecular signatures of organoids are directly influenced by the composition of the synthetic matrices. Notably, according to the Moffitt et al. pancreatic cancer subtype classification [14], synthetic matrices shifted the molecular signature of organoids to a more basal‐like profile relative to Matrigel (Figure 1d). Although the direction of the shift was conserved, the magnitude varied across donors and compositions, underscoring interpatient variability in responses to microenvironmental cues. A donor × composition interaction model supported heterogeneity in response magnitude (Figure S3, Data S2).

Given that the composition of the synthetic matrix dictates the transcriptome in pancreatic cancer organoids, we devised a four‐stage workflow to systematically engineer transcriptional programs through modulating the composition of the synthetic matrices (Figure 1e). First, we performed high‐throughput composition screening across three different pancreatic cancer cell lines representing diverse disease states to identify broadly permissive matrix formulations that support cell growth and viability. Second, we implemented a Design of Experiments (DoE) approach to model transcriptional changes in organoids as a function of C‐F‐L peptide ratios, establishing predictive relationships between synthetic matrix cues and global gene expression. Third, we applied multiobjective optimization to pinpoint matrix compositions that bias predefined transcriptional programs, using EMT as a well‐characterized and clinically relevant benchmark. Finally, we validated this pipeline by culturing pancreatic cancer organoids in the optimized matrices and performing bulk RNA sequencing and secretome profiling, demonstrating the enrichment of EMT‐associated transcriptional and secretory signatures. This integrated screening, modeling, and optimization platform provides a rational strategy for programming cell state transitions in organoids through precise control of microenvironmental signals.

**FIGURE 2:**
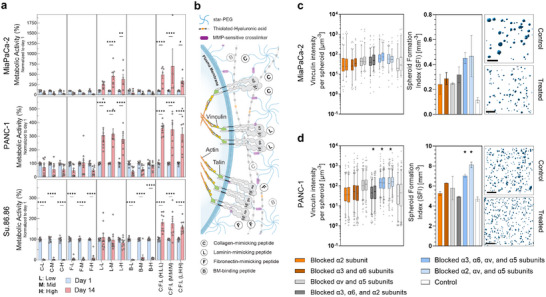
Modulating the synthetic matrix composition directly impacts cell growth and dynamics. (a) Metabolic activity of three phenotypically distinct pancreatic cancer cell lines, demonstrating that multi‐peptide synthetic matrices sustain cell growth over extended culture periods (MiaPaCa‐2 and PANC‐1: *N* = 2 independent biological replicates, *n* = 4 independent experimental replicates each, mean ± SEM, two‐way mixed‐effects RM ANOVA, *p < 0.05, **p < 0.01, ****p < 0.0001; Su.86.86: *N* = 3 independent biological replicates, *n* = 3 independent experimental replicates each, mean ± SEM, two‐way RM ANOVA, ****p < 0.0001). (b) Schematic of integrin α subunits expected to engage C, F, and L peptides. Parts of the schematic were created using BioRender.com. (c,d) Blocking integrin‐mediated cell adhesion in multi‐peptide matrices alters cytoskeletal organization and morphological features in MiaPaCa‐2 (c) and PANC‐1 (d) cultures, evidenced by the change in vinculin expression and decreased spheroid size following functional blocking of different integrin α‐subunits (*N* = 2 independent experimental replicates; box and whiskers: boxes represent the 25th, 50th and 75th percentiles; lower and upper whiskers represent 5–95 percentiles, nested one‐way ANOVA, *p < 0.05; bars: mean ± SEM, one‐way ANOVA, *p < 0.05). Representative segmented pseudo‐colored inserts show spheroids in 3D cultures treated with integrin α2, αv, and α5 blocking cocktails; scale bars 400 µm.

We analyzed metabolic activity and cell adhesion dynamics in various synthetic matrix compositions using high‐throughput analyses. These included three pancreatic cancer cell lines derived from primary tumors (MiaPaCa‐2 and PANC‐1) and liver metastasis (Su.86.86), representing diverse phenotypic traits and invasive profiles. We evaluated the metabolic activity of encapsulated cells in matrices tethered with varying levels of individual or combinations of C, F, L, and B peptides (Figure 2a). The peptide B content was held constant in all multi‐peptide compositions to maintain consistent matrix affinity for cell‐secreted laminins and collagen IV. Mono‐peptide matrices did not support growth except those containing L peptide (with IKVAV as the active sequence), consistent with its known role in promoting cell survival, proliferation, and metabolic activity [20]. The integrin targets in other mono‐peptide compositions are primarily associated with general cell adhesion, motility, and differentiation [7]. In contrast, all multi‐peptide compositions showed robust metabolic activity after 14 days of 3D culture, although the difference between peptide ratios did not result in statistically significant differences in metabolic activities.

We systematically blocked engaging integrin alpha subunits in the L‐only and multi‐peptide matrices to dissect the contribution of specific integrin‐peptide interactions to cell adhesion‐mediated signaling in our platform (Figure 2c,d; Figure S4). Peptide concentrations were set at the midpoint of the experimental design, which was tested in coarse viability profiling of the compositions. Blocking only α2 integrin had little impact on spheroid formation or normalized vinculin expression. In contrast, blocking F‐binding integrin subunits led to reduced spheroid size, yet increased vinculin expression per spheroid. The same trend was observed in L‐only matrices (Figure S4c). As αv and α5 integrin subunits do not engage with the L peptide [7], combined blockade of integrins targeting both F and C peptides further impaired spheroid formation. Combined blockade of integrins targeting both F and C peptides shifted cultures toward a higher number of smaller segmented spheroids (increased spheroid counts and reduced median volumes), consistent with a fragmentation or reduced‐compaction morphology (Figure S5). These observations support the rationale that combining C, F, and L peptides affects cell proliferation, adhesion, and motility, and that their combination may facilitate or prevent downstream cell behavior.

**FIGURE 3:**
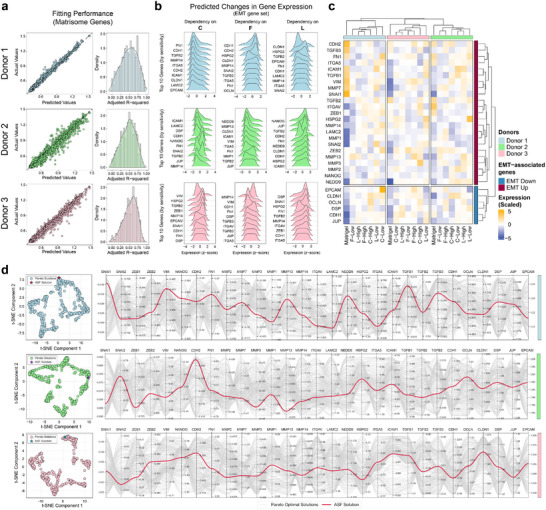
Transcriptomic modeling and multiobjective optimization of ECM‐driven gene expression in pancreatic cancer organoids. (a) Performance assessment of fitted models for matrisome‐associated genes using five out‐of‐sample confirmation compositions sampled within the CCD factor bounds. These confirmation compositions evaluate interpolation within the predefined design region. Scatterplots show predicted vs. actual gene expression values. Adjacent density plots illustrate the distribution of adjusted R^2^ values from the training dataset for each donor. (b) Ridge plots showing predicted expression changes for the top 10 EMT‐associated genes across gradients of individual peptides for each donor. Expression levels were normalized to each gene's predicted value at the median peptide concentration. For each peptide‐specific gradient, other peptides were held constant at their minimal concentrations. (c) Heatmap illustrating the EMT gene expression in selected samples with high or low peptide contents. EMT‐associated genes were categorized based on upregulation or downregulation during EMT. Matrigel‐cultured organoids provide the baseline expression for comparison. (d) Multiobjective optimization results for the EMT‐associated gene set across donors. t‐SNE plots show the clustering and distribution of Pareto‐optimal solutions, highlighting the proximity of the selected Achievement Scalarizing Function (ASF) solutions to these optimal regions. Parallel coordinate plots illustrate normalized gene expression profiles across all candidate solutions, with ASF‐selected solutions highlighted in red, emphasizing their optimized transcriptional profiles.

To harness this modulatory characteristic, we used a DoE approach to grow organoids from three patients in 23 synthetic matrix compositions by combining C, F, and L peptides at three experimental levels, followed by bulk RNA‐sequencing. We used second‐order polynomial fitting to generate expression functions for each gene based on the content of each peptide and their interaction terms. We assessed out‐of‐sample interpolation by comparing predicted vs. observed expression in five confirmation compositions per donor, not used during model fitting and constrained within the CCD bounds (Figure 3a; Table S1). The goodness‐of‐fit across the modeled genes was evaluated by the adjusted R^2^ values computed using the training dataset. The distribution of adjusted R^2^ values for matrisome‐related genes in patients 1 and 3 was asymmetric, with the peak at 55.9% and 61.2%, respectively, while the mode value in patient 2 was 50.3%. We believe two main factors contribute to this broad range of fitting metrics within each donor. First, sequencing technical factors, including library preparation efficiency, sequencing depth, and the input RNA amount for center point samples in the DoE, inevitably impacted the fitted models. To mitigate sample‐level noise due to technical variability, we performed weighted regression in DoE analysis using group‐level weighting. This step minimized sample‐level scaling noise and improved model consistency across donors (Figure S6). Second, not all genes are expected to strongly depend on the extent of expression of cell adhesion molecules, which directly engage with presented peptides in the synthetic matrices.

To illustrate the predictive trends in modeled gene expression, we generated ridge plots showing the top 10 predicted normalized expression responses of genes associated with EMT across individual peptide gradients (Figure 3b). EMT was selected as a representative gene program because of its coordinated regulation and its relevance to phenotypic processes that are sensitive to microenvironmental cues. For this analysis, we included only EMT‐related genes with well‐fitted expression functions (adjusted R^2^ value above 0.5). The resulting profiles revealed a spectrum of peptide sensitivities, with some genes displaying pronounced shifts in expression while others exhibited minimal or noisy trends. This variability underscores that the predictive accuracy of individual expression functions is often moderate at best and possibly insufficient to infer a deterministic outcome alone. Expression levels of the entire gene set in selected samples with high or low peptide levels confirm the observation that subtle changes among different conditions for a given gene are present (Figure 3c). Yet, when multiple such factors collectively influence the system, their aggregated effect can be substantial, nudging the cell state toward distinct transcriptional trajectories associated with phenotypic states even in the absence of sharply predictive single‐gene behavior. Such emergent behavior is consistent with known nonlinear and threshold effects in gene regulation, where cooperative interactions within transcriptional networks can amplify modest upstream inputs into broad transcriptional shifts [21].

We employed a multiobjective optimization algorithm based on NSGA‐II to identify Pareto‐optimal synthetic matrix compositions that maximize or minimize the predicted expression of multiple target genes (Figure 3d). Pareto optimality reflects non‐dominance rather than proximity, and solutions that are Pareto‐optimal or Pareto‐adjacent can remain substantially separated along one or more objectives. Prioritization among solutions was performed using an Achievement Scalarizing Function (ASF), weighting each objective by its adjusted R^2^ values to account for prediction confidence. Although the ASF‐optimal solution lies close to the neighboring Pareto solutions, as evident in the qualitative representation of the Pareto manifold in t‐SNE plots, the predicted transcriptional differences are expected to be consistent with nonlinear amplification and threshold effects commonly observed in regulatory networks when multiple upstream cues are perturbed in combination. To experimentally test this, we selected a control composition for each patient close to the Pareto front overall (maximum 9–12% difference from the Pareto‐optimal solutions) but with a normalized Chebyshev distance of moderate to large (20–60% in objective space) from the ASF‐optimal solution (Data S3). This strategy enabled us to directly examine whether small to moderate predicted shifts across multiple transcriptional targets can induce large‐scale reprogramming of organoid states.

**FIGURE 4:**
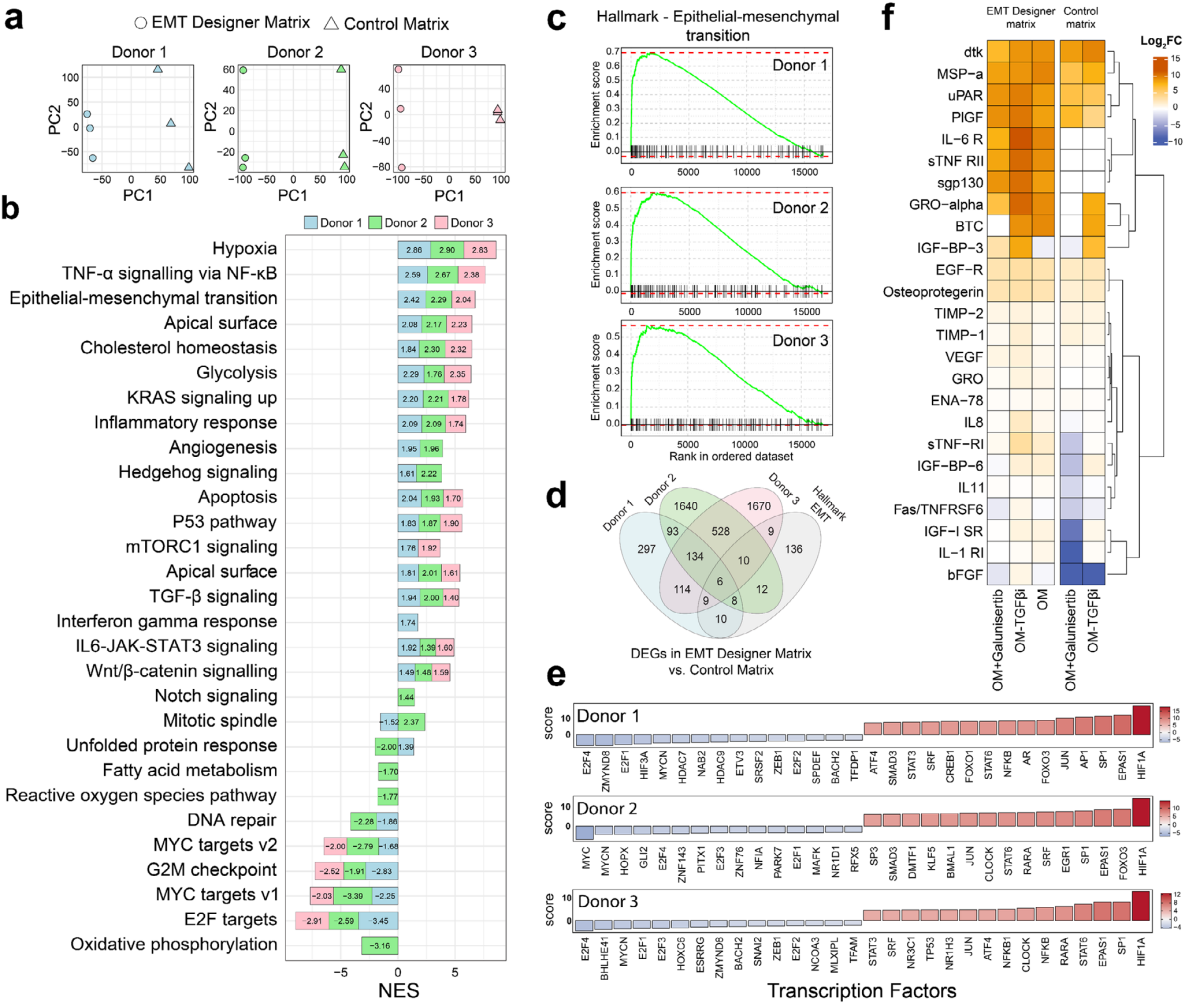
Experimental validation of EMT‐associated transcriptional program enrichment in pancreatic cancer organoids. (a) PCA illustrating transcriptional differences between organoids cultured in the multiobjective optimized matrix (EMT Designer matrix) vs. control matrices across donors. Distinct separations is consistent with bulk transcriptome shift in optimized matrices. (b) Pre‐ranked GSEA showing normalized enrichment scores of hallmark gene sets significantly enriched in organoids cultured in optimal matrices compared with controls across donors. (c) Specific enrichment scores for the EMT hallmark gene set, emphasizing enrichment of EMT‐related transcriptional programs across three donors in optimized matrices. (d) Venn diagram illustrating the overlap between differentially expressed genes (EMT Designer vs. control matrices) and the EMT hallmark gene set, highlighting the specificity and biological relevance of induced transcriptional changes. (e) Transcription factor (TF) activities of organoids cultured in EMT Designer matrix compared with the control for three donor samples. The top 30 TFs for each donor and the corresponding scores are presented. (f) Heatmap displaying secreted protein expression levels of Donor 1 organoids cultured in EMT Designer vs. control matrices. Data presented as the log_2_ fold‐change values of quantified secreted factors for each experimental condition with respect to the organoids cultured in the control matrix in standard organoid medium (OM). The removal of the TGF‐β inhibitor from the culture medium resulted in a substantial increase in secreted factors previously implicated in EMT‐associated signaling at the protein level.

To validate these predictions, we cultured organoids from each patient in the ASF‐selected matrix compositions, hereafter referred to as EMT Designer matrices, alongside matched control matrices. EMT Designer matrix, by definition, refers to a composition that is optimized to enrich the EMT‐associated transcriptomic signatures. PCA revealed that organoids from all three patients exhibited significant separation along the first principal component (PC1) between control and EMT Designer conditions (Figure 4a), indicating bulk transcriptional shifts. Gene ontology (GO) analysis of PC1‐associated differentially expressed genes (Data S4) revealed that upregulated genes were enriched in GO terms associated with ECM remodeling, hypoxia adaptation, energy metabolism, and intercellular signaling. In contrast, downregulated genes were associated with genome maintenance, DNA repair, cell cycle regulation, and chromatin organization. These changes are consistent with EMT‐associated transcriptional programs frequently linked to ECM remodeling and stress‐adaptation pathways, alongside reduced proliferation‐associated programs [22, 23]. Notably, no individual genes were significantly associated with the second principal component (PC2) across all the donors. This further emphasizes that the dominant transcriptional changes distinguishing EMT Designer from control matrices are encapsulated by PC1, highlighting a coordinated shift in gene expression reflective of EMT‐associated biological processes.

To assess proximity to the predicted EMT target state, we compared observed expression values in EMT Designer matrices to model predictions for the EMT objective gene set. Agreement was high across donors (Figure S7, Table S2), and deviations were concentrated among a subset of genes with lower DoE model fit. Additionally, by stratifying the EMT objective genes by adjusted R^2^ and summarizing their differential expression in EMT Designer vs. control matrices, we observed donor‐dependent and non‐monotonic enrichment (Figure S8). Across donors, a substantial fraction of EMT objective DEGs fall into the moderate‐to‐high adjusted R^2^ strata, consistent with the optimization strategy that weights objectives by adjusted R^2^ during ASF‐based selection. At the same time, the donor‐dependent variability indicates that differential expression in EMT Designer matrices is not captured purely by single‐gene predictability metrics, consistent with the expected stochasticity and network‐governed propagation in transcriptional regulation [21].

Consistent with the transcriptional shift, gene set enrichment analysis (GSEA) confirmed enrichment of EMT‐associated programs across all patients using the hallmark gene sets (Figure 4b,c). Among the top enriched pathways were hypoxia, TNF‐α signaling, and EMT. A core set of genes, including *VEGFA*, *ENO2*, *QSOX1*, *ECM1*, and *PLOD2*, was consistently upregulated across all patients (Figure 4d; Data S4), aligning with known gene signatures previously associated with EMT and pancreatic cancer progression [24, 25, 26, 27, 28]. Although a substantially higher patient‐specific expression of key driver genes in EMT was observed (Data S4). The extent of differential expression within the EMT hallmark gene set strongly indicated a convergent enrichment of mesenchymal‐associated modules, consistent with partial EMT‐like reprogramming.

We analyzed the donor‐specific transcription factor (TF) activity in EMT Designer matrices compared with the respective controls (Figure 4e). TFs with high scores in all the donors were associated with hypoxia, inflammatory response, and canonical TGF‐β effectors. In contrast, some epigenetic EMT brakes such as PDEF, ZMYND8, PITX1, and NR1D1 were scored low. This further supports the enrichment of EMT‐associated regulatory programs. We speculate that the observed negative TF scores for some key regulators associated with canonical SMAD‐dependent EMT drivers were due to the blockade of ALK5 in conventional pancreatic organoid medium [29, 30]. Together, these regulatory shifts suggest a matrix‐conditioned EMT‐associated program that does not necessarily imply full epithelial‐to‐mesenchymal conversion.

To assess whether the transcriptional reprogramming was reflected at the protein level, we analyzed the secretome profiles using a cytokine array. Given the central role of TGF‐β signaling in orchestrating EMT [31, 32] and its pharmacological inhibition in pancreatic cancer organoid cultures [29, 30], we compared secretomes using three conditions, including standard organoid medium (OM), OM without the TGF‐β inhibitor A‐83‐01, and OM with Galunisertib, an experimental EMT inhibitor, replacing A‐83‐01. Removal of the TGF‐β inhibitor led to elevated secretion of multiple EMT‐associated factors, including IL‐6R [33], IL‐8 [34], GRO‐α [35], uPAR [36], TIMP‐1 [37], and VEGF [38] (Figure 4f), specifically in organoids cultured in our EMT Designer matrix. Galunisertib inhibited the secretion of key factors in the control matrix, although its effect was more limited due to lower potency and less direct impact on nodal/activin signaling. However, its efficacy was low in the inhibition of the same secreted factors in the EMT Designer matrix, compared with the OM in the control matrix.

Immunofluorescence analysis of organoids cultured in control vs. EMT Designer matrices (Figure S9) revealed matrix‐dependent remodeling of epithelial junction and cytoskeletal organization. Compared with controls, EMT Designer cultures showed reduced continuity or redistribution of E‐cadherin together with altered F‐actin architecture and reduced spatial coupling of these markers, consistent with partial EMT‐like remodeling that was heterogeneous across spheroids and donors.

These findings indicate that the EMT Designer matrices can bias organoids toward a persistent EMT‐like transcriptional and secretory state under standard organoid medium conditions and across soluble‐factor perturbations tested here.

## 3 Discussion

In this study, we describe a rational design strategy to steer transcriptional states in organoid cultures through computationally guided modulation of the synthetic ECM composition. Our results demonstrate that synthetic matrix compositions identified through modeling of the transcriptome and multiobjective optimization can program cancer organoids into distinct transcriptional and secretory states. The observed shift in transcriptional state in response to modest compositional changes in matrix design reflects the inherent sensitivity of cellular regulatory networks to microenvironmental cues. By integrating computational design with experimental validation across independent donors, we demonstrate that quantitative modulation of adhesion cues can bias organoid transcriptional state within a fixed, standardized soluble‐factor background. The demonstrated approach is inherently donor‐specific, since the optimal matrix composition depends on how each donor's transcriptional profile responds to specific ECM components. This relationship stems from the oncogenic mutational profile of the organoids, epigenetic factors, and the genomic stability of the organoids in culture. While this could potentially limit the scalability of this approach for generalized solutions, its power lies in a strong personalized‐medicine approach, where specific transcriptional programs in individual patients can be modelled and interrogated.

A limitation of this study is the small cohort size, given the inherent heterogeneity of the pancreatic TME. While we observed consistent state‐level shifts between EMT Designer and control conditions within each donor, this cohort does not allow estimation of performance variability across the broader heterogeneity of pancreatic cancer. Future work should apply the same workflow across larger organoid biobanks to quantify success rates and variance, and to test whether responsiveness correlates with molecular subtype, mutational background, or baseline pathway activity.

We chose EMT as a proof of concept for two main reasons: its critical role in cancer progression, prognosis, and therapeutic decisions and outcomes; and its susceptibility to microenvironmental cues, owing to its ECM‐centric transcriptional regulators [39]. Because all transcriptomic measurements in this study are bulk RNA‐seq, the observed EMT‐associated shifts represent population‐average changes and cannot distinguish a uniform state shift from redistribution or expansion of EMT‐primed or hybrid epithelial‐mesenchymal subpopulations. Immunofluorescence imaging qualitatively shows heterogeneous junctional and cytoskeletal remodeling across spheroids, suggesting that the EMT Designer matrix biases cell‐state distributions rather than enforcing a homogeneous transition. In our results, we observed a substantial shift in transcriptional patterns of organoids cultured in EMT Designer matrices, despite ALK5 blockade via OM supplements. The persistence of EMT‐associated transcriptional programs despite ALK5 inhibition suggests the possibility of ECM‐mediated activation of latent TGF‐β or alternative EMT‐driving pathways. While we did not attempt to decouple or co‐optimize all soluble niche components, our data show that matrix composition can bias EMT‐associated transcriptional programs even under the standardized organoid medium used here.

In organoid systems, soluble factors and ECM cues are integrated rather than treated as independent: soluble ligands control pathway availability, whereas ECM engagement regulates receptor organization, downstream mechanotransduction, and the local presentation/activation of growth factors. In particular, integrin‐mediated activation of latent TGF‐β provides a plausible route by which ECM context can modulate TGF‐β family signaling even when canonical receptor signaling is pharmacologically perturbed [40, 41, 42]. Importantly, in this study, we treated organoid medium as a controlled baseline and asked whether defined adhesive cues can bias cell state within that baseline. This framing aligns with general organoid guidance, which emphasizes that matrix and soluble factor selection jointly determine phenotype and experimental reproducibility [43, 44, 45]. Consistent with this context dependence, baseline pathway enrichment differs between Matrigel as the standard culture ECM and the defined PEG matrices (Figure S10), underscoring that the cross‐ECM comparisons primarily reflect distinct microenvironmental regimes rather than a single efficiency scale. A practical implication is that the apparent magnitude of ECM effects will depend on the soluble‐factor regime (for example, highly saturating soluble conditions may compress the dynamic range of matrix‐driven differences). Future extensions of this platform could incorporate selected soluble factors as additional design variables to co‐optimize ECM and media for specific biological questions.

Our findings underscore the utility of synthetic matrices as active programming inputs that leverage established biological mechanisms. In the context of EMT, extensive literature has characterized the specific signaling axes linking integrin engagement to EMT drivers [46]. Rather than deconstructing the driver axes via reductionist approaches, our platform adapts a complementary systems‐engineering framework. By modelling the combinatorial integration of the known adhesive cues, we demonstrate that cell states can be rationally steered toward complex phenotypic outcomes. This approach shifts the focus from identifying causal nodes to exploiting and optimizing collective signaling inputs required to overcome homeostatic constraints.

We showcase the implementation of this approach using pancreatic cancer organoids, which represent a highly desmoplastic and ECM‐rich microenvironment. As ECM‐originated signaling through integrins is central in our proposed approach, we hypothesized that a similar ECM‐guided approach may be applicable to other ECM‐rich TMEs characterized by integrin‐mediated signaling, such as non‐small cell lung cancer [47], triple‐negative breast cancer [48], ovarian cancer [49], or bone metastatic microenvironment, although further experimental validation is warranted. Taken together, we envision that this rational design strategy establishes a framework with tunable design parameters for bottom‐up engineering of tissue microenvironments to control complex cell states, with broad implications for regenerative medicine, cancer biology, and biomaterial design.

## 4 Conclusion

In this study, a computationally guided strategy is established to bias organoid transcriptional state through bottom‐up engineering of a defined synthetic ECM. By combining high‐throughput screening, a DoE‐based transcriptomic model of matrix‐presented adhesion cues, and multiobjective optimization, donor‐specific matrix compositions are identified that reproducibly shift pancreatic cancer organoids between distinct bulk transcriptional and secretory states under a standardized soluble‐factor background. Using EMT as a proof‐of‐concept benchmark, the optimized “EMT Designer” matrices consistently enrich EMT‐associated gene sets across donors, alongside coordinated changes in pathways linked to ECM remodeling, hypoxia, inflammatory signaling, and altered proliferative programs. Evidence from transcriptome and protein‐level expression is consistent with a partial EMT‐like phenotype. Overall, these results support the view that minimal, defined adhesion cues can function as programmable inputs to steer complex transcriptional programs in patient‐derived organoids.

## 5 Experimental Section

### 5.1 Conditioned Mediums

The triple Wnt/R‐Spondin/Noggin (WRN)‐conditioned medium was produced using L‐WRN cells (ATCC, CRL‐3276) based on a modified version of a published protocol [50]. Briefly, L‐WRN cells were cultured and passaged in high glucose DMEM with GlutaMAX supplement (Gibco) with 10% (v/v) fetal bovine serum (FBS, Sigma–Aldrich), with G‐418 and hygromycin B (InvivoGen, 0.5 mg/mL each). For conditioned medium production, cells were grown until confluency in the culture medium without G‐418 and hygromycin B. The medium was then changed to Advanced DMEM/F12 medium (Gibco) with 10 mM HEPES (Gibco), 1X GlutaMAX (Gibco), 1% Penicillin‐Streptomycin (PS, Sigma–Aldrich), and 20% FBS. The medium was replaced and collected every 24 h for four collections. The collected medium on each day was centrifuged at 2000 × g, sterile filtered, and stored at 4°C. The pooled collection was mixed with an equal volume of fresh medium without FBS, aliquoted, and stored at −20°C until use. WNT3a‐, RSPO1‐, and mNoggin conditioned media were produced using HEK293 cell lines from Hubrecht Institute (Utrecht, Netherlands).

### 5.2 Ethical Approval and Consent to Participate

Ethical approval for the use of patient‐derived samples was granted by the Department of Visceral, Thoracic, and Vascular Surgery at TU Dresden's University Hospital Carl Gustav Carus (Ethics Committee Dresden, approval number EK76032013). All patients and donors gave informed consent. The study adhered to the ethical guidelines outlined in the Declaration of Helsinki.

### 5.3 Human Pancreatic Cancer Organoid Culture

Human pancreatic cancer organoids were provided by the laboratory of D. E. Stange (TU Dresden) as part of a collaboration agreement. Organoid cultures were routinely tested for mycoplasma contamination. Organoids were maintained in human Organoid Medium (OM) composed of Advanced DMEM/F12 supplemented with 10 mM HEPES, 1X GlutaMAX, and 1% PS as the base medium. The base medium was supplemented with either WRN‐conditioned medium (50% v/v) or WNT3a‐conditioned medium (50% v/v) plus RSPO1‐ and mNoggin‐conditioned media (10% v/v each), and further supplemented with 1X B27 (Gibco), 1X N2 (Gibco), human gastrin I (1 nM, Sigma–Aldrich), N‐acetyl‐L‐cysteine (1 mM, Sigma–Aldrich), nicotinamide (10 mM, Sigma–Aldrich), recombinant human EGF (50 ng/mL, PeproTech), recombinant human FGF‐10 (100 ng/mL, PeproTech), and A‐83‐01 (0.5 µM, Tocris Bioscience).

Routine organoid culture and expansion were performed in the basement membrane secreted by Engelbreth‐Holm‐Swarm mouse sarcoma (Matrigel, growth‐factor reduced, Corning). Organoids were expanded in 50 µL Matrigel domes for up to 2 weeks before passaging, with medium renewal every other day. Organoid cultures were passaged by mechanical dissociation of the domes using vigorous pipetting through fine pipette tips and plating in new Matrigel domes with the split ratio between 1:2 and 1:4. Complete OM was supplemented with Rho‐kinase inhibitor Y‐27632 (10 µM, Selleck Chemicals) during the first 48 h after each passaging. The WRN‐conditioned medium was only used to expand organoids. The conditioned media containing the individual niche factors were used for the experiments.

### 5.4 Human Pancreatic Cancer Cell Lines

Cells were grown at 37°C in humidified air supplemented with 5% CO_2_. Cultures were tested routinely for mycoplasma contamination. The human pancreatic cancer cell line MiaPaCa‐2 was obtained from the Deutsche Sammlung von Mikroorganismen und Zellkulturen (DSMZ) in Braunschweig, Germany. The human pancreatic cancer cell lines Su.86.86 and PANC‐1 were purchased from ATCC. MiaPaCa‐2 and PANC‐1 cells were maintained in high‐glucose DMEM with GlutaMAX supplement (Gibco) with 10% (v/v) FBS and 1% PS. Su.86.86 cells were cultured in RPMI 1640 (PAN‐Biotech) supplemented with 10% (v/v) FBS and 1% PS. Culture media were changed twice weekly, and all the cell lines were passaged upon reaching 80–90% confluency using 1X TrypLE Express Enzyme (Gibco).

### 5.5 Macromers and Synthetic Peptides

Star‐shaped 8‐arm PEG vinyl sulfone macromers (PEG‐VS, 20 kDa) were purchased from JenKem Technology. Thiolated‐hyaluronic acid (HASH, 1 MDa, degree of substitution 5.16 mol%) was purchased from Creative PEGWorks. A 4‐arm star‐shaped PEG (PEG‐MMP) conjugated with MMP‐cleavable peptide sequence (Ac‐CGGPQGIWGQGGCG) was produced in‐house [51]. The following ECM‐mimicking peptides were purchased from Activotec (Cambridgeshire, UK) with a minimum of 95% purity, with the active adhesion sites including: GFOGER, a triple‐helical Collagen type I derived sequence (Ac)GGYGGGPG(GPP)_5_GFOGER(GPP)_5_GPC(Am) labeled as the letter “**C**”; IKVAV, part of the sequence derived from the α1 chain of Laminin‐1 (Ac)CSRARKQAASIKVAVSADR(Am) labeled as the letter “**L**”; a sequence derived from nidogen‐1 with binding affinity with basement membrane secreted proteins (Ac)GCREISAFLGIPFAEPPMGPRRFLPPEPKKP(Am) labeled as the letter “**B**”. The peptide labeled with the letter “**F**” (PHSRNGGG‐K‐[GGC(Ac)]‐GGRGDSPY(Am)) was purchased from CASLO ApS (Kongens Lyngby, Denmark) with a minimum purity of 95%. It combines the PHSRN synergy site and the RGD adhesion motif into a branched structure derived from domains 9–10 of fibronectin type III. All the peptides were reconstituted in MilliQ water.

### 5.6 Synthetic Hydrogels Preparation

HASH content was set to 0.1% of total PEG solid content in each hydrogel formulation and was dissolved in 0.3 M HEPES (Merck Millipore). The final concentrations of C peptide ranged from 0.75 to 1.5 mM. For F, L, and B peptides, the content for each peptide ranged from 0.15 to 0.375 mM. Hydrogels were prepared using a two‐step sequential conjugation and crosslinking process. The total PEG content determined the total concentration of hydrogels. In the first step, HASH and the desired peptides were conjugated to PEG‐VS dissolved in 0.3 M HEPES. The conjugation order was HASH, C, F, L, and B. This order was maintained for every preparation. A reaction time of 15 min was given to complete the conjugation in every addition step. In the second step, the pre‐conjugated PEG‐VS was thoroughly mixed with cells suspended in a solution of PEG‐MMP in PBS at neutral pH. The mixture was then plated into low‐attachment 96‐well plates, either manually or using a liquid handling workstation for high‐throughput experiments (JANUS G3, PerkinElmer). Samples were incubated at 37°C for 10 min before being overlaid with culture medium. All precursor solutions were freshly prepared immediately before use. The conjugation ratio (δ), defined as the number of conjugated arms per PEG‐VS molecule by peptides, varied between 0.25 and 0.375 for the stiffness screening experiment. All cell‐containing experiments described in this manuscript used a δ value of 0.375.

### 5.7 Cell Viability Measurements

Cell viability in 3D cultures was assessed using the alamarBlue assay (Invitrogen). In brief, assay media were prepared by 1:25 dilution of 10X stock in high‐glucose DMEM with L‐glutamine and 25 mM HEPES without phenol red (Gibco) for cell line cultures. Cultures were incubated in assay media at 37°C in humidified air supplemented with 5% CO_2_ for 6 h. After incubation, the supernatants were collected, and fluorescence was measured using a Spark microplate reader (TECAN) at 560 nm/590 nm excitation/emission. Supernatants from acellular hydrogels were used as blanks under the same conditions.

### 5.8 Integrin Function‐Blocking Treatments

Integrin α‐subunits were blocked in 3D using the following function‐blocking antibodies purchased from BioLegend: anti‐α2 (clone P1E6‐C5, 5 µg/mL), anti‐α3 (clone P15B, 5 µg/mL), anti‐α5 (clone NKI‐SAM‐1, 5 µg/mL), anti‐α6 (clone GoH3, 5 µg/mL), and anti‐αv (clone NKI‐M9, 5 µg/mL). Cultures were treated daily with freshly prepared medium containing either individual antibodies or combinations thereof for 7 days, starting from Day 2 of culture.

### 5.9 Immunofluorescence staining of 3D cultures

Hydrogel samples were washed twice in PBS before fixation in 4% PFA in PBS (Thermo Fisher Scientific) for 20 min at 4°C. After fixation, samples were washed twice using PBS for 10 min and permeabilized using 0.1% Triton X‐100 (Sigma–Aldrich) for 20 min at room temperature. Following a single PBS wash, samples were blocked with 2% BSA (Sigma–Aldrich) in PBS for 3 h at room temperature. Cells were stained using primary mouse anti‐vinculin (1:200, Sigma–Aldrich, catalog no. V9264) or primary mouse anti‐E‐cadherin (1:100, BD Bioscience, catalog no. 610182) for 72 h at 4°C. Samples were washed with 0.1% BSA in PBS three times and incubated with Alexa Fluor 488 or 568 conjugated goat anti‐mouse secondary antibody (1:200, Invitrogen, catalog no. A‐11001, A‐11004) overnight at 4°C. Samples were washed with 0.1% BSA in PBS three times with 15 min incubations, and stained for F‐actin and nuclear DNA for 1 h at room temperature using Phalloidin ATTO 633 (1:400, ATTO‐TEC GmbH, catalog no. AD 633) and DAPI (5 µg/mL, Sigma–Aldrich, catalog no. D8417), respectively. Samples were washed with PBS thrice with 15‐min incubations before imaging.

### 5.10 Confocal Microscopy and High‐Content Image Acquisition and Analysis

Hydrogels cultured in uncoated glass‐bottom µ‐Plate 96‐well plates with round wells (ibidi) were imaged using the confocal high‐content screening system Opera Phenix (PerkinElmer). A 5X air objective (NA 0.16, WD 12.1 mm) for brightfield imaging or a 20X air objective (NA 0.4, WD 8.28 mm) for fluorescence imaging was used. Brightfield z‐stacks were captured with a 20 µm distance between planes. Fluorescence z‐stacks were captured using a 3.0 µm plane distance over a total height of 450 µm. Images were acquired with 2% overlaps between adjacent fields. Using Harmony 5.0 (PerkinElmer), raw images were exported as TIFF and stitched into planar mosaics using a Python script. Linear blending was used for overlapping regions. Stitched mosaics were stacked using Imaris File Converter 10.2.0 (Oxford Instruments). Generated z‐stacks were analyzed using Imaris 10.2.0 (Oxford Instruments). To quantify the vinculin expression in each sample, batch image processing was performed to quantify the formed spheroids, the number of nuclei per spheroid, and the volumetric intensity of vinculin in each spheroid. The Spheroid Formation Index (SFI) was calculated using Equation (1), where *N_s_* is the number of spheroids, $V_{s}^{med}$ is the median spheroid volume, and *n* is the total number of nuclei:

(1) $$SFI=\frac{N_{s}}{V_{s}^{med}\times n}$$

With these definitions, higher SFI corresponds to more numerous and/or smaller segmented spheroids per total nuclei (fragmentation or reduced compaction), whereas lower SFI corresponds to fewer and/or larger spheroids per nuclei. Vinculin density was defined as the total vinculin signal per spheroid, normalized to the spheroid's volume.

Confocal microscopy was performed using a Dragonfly Spinning Disc confocal microscope (Andor Technology Ltd.) using a 40X water objective (NA 1.15, WD 0.60 mm) with a spatial resolution of 0.3 µm.

### 5.11 Atomic Force Microscopy (AFM)

AFM indentation measurements were performed using a Nanowizard I AFM (JPK Instruments) mounted on an Axiovert 200 inverted microscope (Zeiss). Hydrogels were incubated in the culture medium for 24 h before indentation to reach the equilibrium swelling. Samples were immersed in PBS at room temperature during the measurements. A 5 µm diameter polystyrene microsphere (Microparticles GmbH) was fixed on a tip‐less cantilever (PNP‐TR‐TL, NanoAndNore GmbH) with the nominal spring constant of 0.08 N/m using a two‐component epoxy glue (Araldite). A calibration step using the thermal noise method was performed prior to the measurements. For each indentation map, a region of interest (ROI) consisting of 7 × 7 points with a spacing of 11.43 µm was probed. The approach velocity and maximum force were set to 5 µm/s and 5 nN, respectively. The Herz model for a spherical indenter was used to fit the force‐distance curves to obtain Young's modulus. Data were processed using JPK data processing software (JPK Instruments). At least three different ROIs were mapped for each sample.

### 5.12 Design of Experiments (DoE)

A DoE approach based on Response Surface Methodology was used to investigate the individual and interaction effects of peptide content on the transcriptional profiles of organoids from three donors cultured in synthetic matrices. A central composite design (CCD) with nine center points was implemented. The molar concentrations of each peptide were varied at three levels (high, center, and low). For peptide C, the corresponding levels were 0.75, 1.125, and 1.5 mM. The corresponding levels for peptides F and L were 0.15, 0.2625, and 0.375 mM. Experimental conditions were randomized to minimize systematic bias. The content of peptide B was kept constant at 0.2625 mM for all experiments. Five random sets of (C, F, L) molar concentrations within the experimental range were selected to validate the generated models (Table S1). These five compositions were used as out‐of‐sample confirmation runs and were not included in model fitting or stepAIC selection.

### 5.13 RNA Isolation

Hydrogels were washed twice with PBS containing Ca^2+^ and Mg^2+^ (Pan Biotech), followed by selective degradation of MMP‐cleavable peptides using 500 U/mL collagenase type I (Gibco) for 45 min at 37°C. Isolated organoids were centrifuged at 600 × g at 4°C for 5 min, resuspended in PBS, and centrifuged again under the same conditions. Organoids from 6–8 independently cultured hydrogel samples per experimental group were pooled for RNA isolation. Total RNA was extracted using the RNeasy Plus Micro Kit (Qiagen) following the manufacturer's protocol. Purified RNA was resuspended in RNase‐free water, aliquoted, and stored at −80°C until use.

### 5.14 Cytokine Profiling

Cell culture media were collected fresh and used without further dilution. The Human Cytokine Array C7 (RayBiotech) was used to profile secreted factors with 60 capture antibodies. Each array membrane was incubated with 1 mL of culture medium overnight at 4°C, and bound antigens were detected according to the manufacturer's protocol. Chemiluminescent signals were captured using a myECL Imager (Thermo Fisher Scientific). Spot intensities were quantified using the analysis spreadsheet provided by the manufacturer.

### 5.15 RNA Sequencing Library Preparation

RNA sequencing libraries were prepared using a standard poly(A)‐enrichment protocol. Briefly, total RNA samples were subjected to mRNA enrichment using oligo(dT) beads, followed by mRNA fragmentation and random hexamer priming. First‐ and second‐strand cDNA synthesis was performed, and the resulting cDNA underwent end repair, 5′ phosphorylation, and dA‐tailing. Indexed adapters were ligated, and libraries were amplified by PCR. The final libraries were quality‐checked, quantified, and sequenced on an Illumina NovaSeq platform with paired‐end 150 bp reads (PE 2 × 150 configuration). Three replicates of each experimental group for each donor were sequenced to account for technical variability.

### 5.16 Bioinformatics and Statistics

Unless otherwise noted, all computational analyses were performed using R version 4.4.1. Heatmaps were generated using the ComplexHeatmap R package (v. 2.20.0). Gene expression data from tumor and normal tissues were obtained via the GEPIA2 [52] web‐based platform using the matched TCGA normal and GTEx data.

The RNA sequencing reads were trimmed using Trimmomatic (v. 0.36) to remove adapter sequences and low‐quality nucleotides. Trimmed reads were aligned to the Homo sapiens GRCh38 reference genome (ENSEMBL) using the STAR aligner (v. 2.5.2b) [53] to generate BAM files. Unique gene hit counts were computed using featureCounts from the Subread package (v.1.5.2). For each donor, only genes with the sum of raw counts more than 10 in at least six samples were included. Variance‐stabilizing transformation (VST) and sequencing depth‐normalization were performed using the DESeq2 [54] (v. 1.44.0). Differential expression between conditions was assessed using Wald tests, and genes with the absolute log_2_ fold change above 0.58 and Benjamini–Hochberg adjusted P value of less than 0.05 were considered significant.

To assign molecular subtype scores according to Moffitt's classification [14], VST counts of basal‐like and classical signature genes were used. Subtype scores were calculated by subtracting the mean VST counts of classical genes from that of basal‐like genes. Negative scores were classified as classical, and the positive scores as basal‐like subtypes. For baseline anchoring, Δ Scores were computed relative to Matrigel within each donor.

PCA was performed using VST counts after excluding genes with zero variance. Gene loadings on principal components (PCs) were used to infer coordinated expression patterns. Significance of associations between genes and PCs was assessed via permutation testing, in which sample labels were shuffled to generate a null distribution of gene‐to‐PC correlations. Empirical p‐values were adjusted using the Benjamini–Hochberg method. Genes with adjusted p‐values below 0.25 were significantly associated with a given PC.

For modeling gene expression, we used reference gene normalization to minimize residual variability attributable to differences in sequencing depth across samples. The reference gene was selected by calculating the coefficient of variation (CV) of each gene using the VST counts across all donors. *CIAO1* was identified as the most stable gene with a CV value of 0.006281975. Expression values of all genes were normalized by dividing by the VST value of *CIAO1* within each sample, followed by averaging across technical replicates for each experimental group.

Reference‐normalized VST counts were used to construct gene expression models based on the DoE design. Quadratic regression models were independently fitted for each gene to capture second‐order and pairwise interaction terms among the three factors (molarity of each peptide C, F, and L). To mitigate the influence of overrepresented center points in the CCD design and the associated impact of sequencing depth variability, weights inversely proportional to the number of replicates per condition were applied during model fitting. Model selection was performed using backward stepwise regression based on the Akaike Information Criterion (AIC), implemented via the stepAIC() function from the MASS R package (v. 7.3.65).

Multiobjective optimization was carried out using the pymoo library (v. 0.6.1.3) in Python (v. 3.12.7). The optimization aimed to simultaneously maximize the expression of upregulated genes and minimize the expression of downregulated genes within a defined biological process, using predictive gene expression models. Peptide molarities served as decision variables, constrained within the design space defined by the DoE. The objective space consisted of predicted expression values of the gene set of interest. A driver gene set comprising key transcriptional regulators and epithelial core components of EMT (Table S3) was curated based on the common consensus in the literature and used in the multiobjective optimization workflow. Optimization was performed using the NSGA‐II algorithm with simulated binary crossover (probability = 0.9, *η* = 15) and polynomial mutation (probability = 0.5, *η* = 20). Population size was scaled to the number of objectives. From the resulting Pareto front, an optimal solution was selected using Achievement Scalarization Function (ASF) decomposition with weights derived from the models’ reliability. Objective values were normalized using estimated ideal and nadir points. User‐defined peptide combinations were evaluated by computing the Chebyshev distance to the optimal solution in normalized objective space. The t‐distributed stochastic neighbor embedding (t‐SNE) was performed using the scikit‐learn implementation, with the perplexity factor set to 50.

GSEA was performed using preranked gene lists created by DESeq2. Pairwise differential expression contrasts between groups were computed using the Wald tests, and genes were ranked according to their corresponding statistics. Normalized Enrichment Scores (NES) were computed for each donor using the GSEA software [55] (v. 4.3.3) using the MSigDB hallmark gene set collection [56]. The fgsea R package (v. 1.30.0) was used to generate the enrichment plots. The maximum and minimum set size parameters in both implementations were set to 500 and 15, respectively. Gene sets with FDR less than 5% were considered significantly enriched.

For the analysis of TF activities, the Wald test scores for differential contrasts from the DESeq2 pipeline were used. The TF collections and their transcriptional targets were derived from the CollecTRI network [57]. Filtered transcription factors with more than 20 target genes were used. Univariate linear modelling from the decoupleR R package (v. 2.10.0) was used to estimate the TF activity [58].

Gene Ontology (GO) term pathway analysis was performed using the enrichGO() function of the clusterProfiler R package (v. 4.12.6), with Benjamini–Hochberg correction for multiple comparisons and the adjusted p‐value cut‐off set to 0.05. The minimum gene set size was set to 10.

GraphPad Prism 10 (GraphPad Software v. 10.4.2) was used to visualize the data from AFM, metabolic activity, vinculin expression, and spheroid formation analysis. Statistical tests included one‐way ANOVA with Tukey's post hoc test for AFM data; mixed‐effect model or two‐way ANOVA with Sidak's post hoc test for metabolic activity; nested one‐way ANOVA with Dunnett correction for vinculin expression; and Welch/Brown‐Forsythe one‐way ANOVA with Dunnett T3 correction for SFI analysis.

RNA sequencing was performed on organoids from 3 donors, with three technical replicates per experimental condition. The metabolic activity of MiaPaCa‐2 and PACN‐1 cells was assessed in two biological replicates per matrix composition, with four technical replicates per condition. The metabolic activity of Su.86.86 cells was analyzed in three biological replicates per matrix composition, each with three technical replicates. Due to the excessive outgrowth of MiaPaCa‐2 cells, 1–2 technical replicates on Day 14 in some groups were excluded to avoid skewing metabolic activity data. Integrin‐blocking experiments were performed with three technical replicates per treatment group. Quantification of vinculin expression and spheroid formation dynamics was based on full‐thickness 20X images acquired from whole hydrogel samples with two replicates per treatment group. Cytokine profiling was performed on Donor 1 samples. For each condition, culture medium from four replicates was pooled. The reported intensity of each cytokine was the average of the normalized signal from two associated spots on the membrane.

## Author Contributions

A.N. and D.L. designed the research. A.N. and D.P. conducted experiments. A.N. analyzed data. V.J.K and D.P. provided technical support. F.B. and D.E.S. provided organoids and reagents. A.N. wrote the manuscript. C.W. and D.L. oversaw the project. D.L. acquired funding and managed the project. All authors read and approved the final manuscript.

## Conflicts of Interest

A.N. and D.L. are listed as inventors in a patent application based on the methodology used in this study.

## Supporting information




**Supporting File 1**: adma72997‐sup‐0001‐SuppMat.docx.

**Supporting File 2**: adma72997‐sup‐0002‐Data.zip.

## Data Availability

The data that support the findings of this study are available from the corresponding author upon reasonable request.
