# Supplementary material for: Bottom‐Up Programming of Cell States in Cancer Organoids with Defined Synthetic Adhesion Cues
Source: Adv Mater. 2026 Apr 14;38(27):e17390. doi: 10.1002/adma.202517390 (PMC13173398; doi:10.1002/adma.202517390)
Supplement: Supplementary file 1 — Supporting File 1: adma72997‐sup‐0001‐SuppMat.docx. [file ADMA-38-e17390-s002.docx]

Supporting Information

**Bottom-Up Programming of Cell States in Cancer Organoids with Defined Synthetic Adhesion Cues**

*Ali Nadernezhad, Verena J. Kast, Dagmar Pette, Franziska Baenke, Daniel E. Stange, Carsten Werner, Daniela Loessner**

**Supporting Note 1: A Modular Roadmap for Guiding Organoid State Transitions**

In organoid research, it is common to observe how individual factors (e.g., specific growth factor) influence a phenotype. However, biological states are typically the results of integrated signals from multiple cues. This workflow provides a methodology for moving from simple observation to the targeted modulation of cell states. The platform suggests candidate regimes that bias the cells toward a desired transcriptomic or phenotypic transition.

**Step 1: Defining the Experimental Space (Adjustable Parameters)**

The first step is identifying the variable inputs (the “parameters”) to be studied. In this work, we focused on three synthetic adhesion peptides that represent a reductionist sample of pancreatic cancer microenvironment. For a general organoid researcher, these parameters could be from a wide range of:

- **Biochemical cues.** Examples: concentration ranges of bioactive chemicals, small molecule inhibitors, or cytokines.
- **Biophysical cues.** Examples: matrix stiffness, hydrogel degradability, or oxygen levels.
- **Cellular cues.** Examples**:** the ratio of different cell types in a co-culture setting.

There are a few considerations when selecting parameters that could significantly impact the rest of the workflow:

- **Relevance and significance:** Selected parameters must be meaningfully relevant and should have a significant impact on the response. The relevant parameters and their significance are usually determined using screening experiments.
- **Operating ranges:** The upper and lower bounds of the parameters must be broad enough to capture interactions, but narrow enough to allow low-degree polynomial models to approximate the true response accurately.
- **Independence:** Parameters should be independent variables that can be manipulated without automatically changing others, ensuring that interaction effects can be measured accurately.

**Step 2: Strategic Mapping (Design of Experiments)**

Characterizing how three to five parameters interact is logistically challenging using traditional methods. For example, testing five parameters at three concentrations each would require 243 different conditions.

Conventional experimentation mainly relies on One-Factor-at-a-Time (OFAT), which works well for studying a few parameters at a limited scale. Instead of using OFAT, we use Design of Experiments (DoE) to efficiently yield a structured subset of about 20-30 representative combinations. In this study, 23 compositions were sufficient to fit second-order (quadratic) trends and interactions across the cues. This allows the researcher to capture synergistic effects, in which one parameter induces a response only when another is present at a specific level, providing a broader view of the experimental landscape with fewer samples.

**Step 3: Quantitative Readouts (State Assessment)**

Once the organoids are cultured in these mapped conditions, the resulting biological state is measured. While this study uses RNA sequencing to assess biological state, the workflow is agnostic to the readout and compatible with any quantitative readout that reflects the state of the organoid. Some examples are:

- **Imaging:** morphological features, branching complexity, or fluorescent intensity of a reporter.
- **Functional:** Metabolic activity, contractility, or drug resistance.
- **Secreted Factors:** Cytokine levels or metabolite production.

Although we only validated transcriptomic readouts here, the workflow is conceptually compatible with other quantitative phenotypes, provided they vary reproducibly across the design space and have sufficient sensitivity to detect interaction effects.

**Step 4: Creating a Predictive Landscape (Modeling)**

The experimental data are used to construct a Predictive Response Surface. This acts as a statistical map of how the inputs correlate with the measured outputs. It is important to note that this model identifies correlations between the matrix and the cell state; it does not inherently prove the underlying molecular mechanism. Instead, it predicts which combinations are most likely to yield a specific biological result, even for conditions that were not explicitly tested in the laboratory. Predictions are most reliable within the tested parameter ranges; using the model to extrapolate beyond them is not recommended without additional data.

**Step 5: Targeted Condition Selection (Multiobjective Optimization)**

Finally, the researcher defines a "Target State." Often, these goals involve trade-offs. This step involves using a scoring system to find a balance. For example, identify a condition that increases a specific marker while maintaining cell viability. The model identifies the “sweet spots” where both criteria are best satisfied. Each criterion is defined as an “objective”, and by exploring the design space, the combinations of parameters that maximize or minimize multiple objectives are identified.

- **The EMT Example:** In this study, we sought to identify matrix compositions that promote mesenchymal gene expression while reducing epithelial markers. In practice, we set 23 genes as 23 objectives to be maximized (markers upregulated during EMT) and 6 genes as 6 objectives to be minimized (markers associated with the epithelial phenotype).

The optimization algorithm searches the predictive landscape from Step 4 to identify the optimal regimes, or simply the specific combinations of inputs, that are predicted to favor this transition. Several algorithms are available for this purpose. The result is typically a family of ‘best-compromise’ conditions (a Pareto set), from which one can be selected based on practical priorities (e.g., viability, cost, ease of implementation) and then experimentally confirmed.

**Summary for the Practitioner**

This roadmap provides a systematic way to identify the microenvironmental conditions that promote specific cellular states. By using a data-driven approach to map the relationship between matrix cues and organoid responses, researchers can identify favorable environments for maturation, disease modeling, or drug testing. While the platform identifies the "what" (the optimal conditions for a transition), it also provides a robust starting point for subsequent studies to investigate the "how" (the specific molecular mechanisms) behind these shifts.

**Supporting Note 2: Glossary of Terms**

**Parameter (input/factor)**

A controllable experimental variable you set in advance (e.g., peptide density, stiffness, oxygen). In this study, the primary parameters were the levels/ratios of matrix-presented adhesion peptides.

**Design space**

The full range of parameter values you decide to explore (for example, “low-medium-high” levels of each peptide). Model-based conclusions are intended to apply within this space, not necessarily beyond it.

**Interaction effect (synergy/dependency)**

A situation where the effect of one parameter depends on the level of another (e.g., a phenotype changes only when cue A is high and cue B is low). This is a key reason to use DoE rather than OFAT.

**Design of Experiments (DoE)**

A structured strategy for choosing a limited set of experimental conditions that still lets you estimate the effects of each parameter and their interactions. In the manuscript, this was implemented using a response-surface DoE (central composite design) over three peptide cues.

**Response surface/predictive landscape**

A fitted statistical map that relates inputs (parameters) to outputs (measured readouts). It provides predicted outcomes for combinations that were not directly tested, but it is correlational rather than mechanistic.

**Quadratic (second-order) model**

A common response-surface model that captures (i) individual cue effects, (ii) pairwise interactions, and (iii) curvature (nonlinear trends) within the tested range. In the manuscript, gene-wise quadratic regressions were used to model expression as a function of peptide levels and interactions.

**Objective (optimization criterion)**

A single quantity you want to increase or decrease (e.g., “maximize mesenchymal markers,” “minimize epithelial markers,” “maintain viability”). In the EMT case, each gene target can be treated as a separate objective.

**Multiobjective optimization**

A method for selecting input conditions that perform well across multiple objectives simultaneously, acknowledging trade-offs rather than assuming one metric captures success. In the manuscript, this was used to prioritize peptide combinations predicted to shift gene programs.

**Trade-off / Pareto-optimal (Pareto front)**

A set of “best-compromise” solutions, where improving one objective would worsen at least one other objective. Practically, it provides a shortlist of candidate conditions rather than a single universally “best” condition.

**Solution selection (scalarization)**

A rule for choosing one candidate from a set of trade-off solutions by weighting objectives according to priorities (in the manuscript, confidence in predictions). In the manuscript, an Achievement Scalarizing Function (ASF) was used to select a representative solution near the Pareto-optimal region.

**Confirmation (Out-of-sample testing)**

An internal check of predictive performance by comparing model predictions to results from conditions not used to fit the model. In the manuscript, a subset of randomly selected matrix compositions was used to assess predicted vs. observed expression for selected genes.


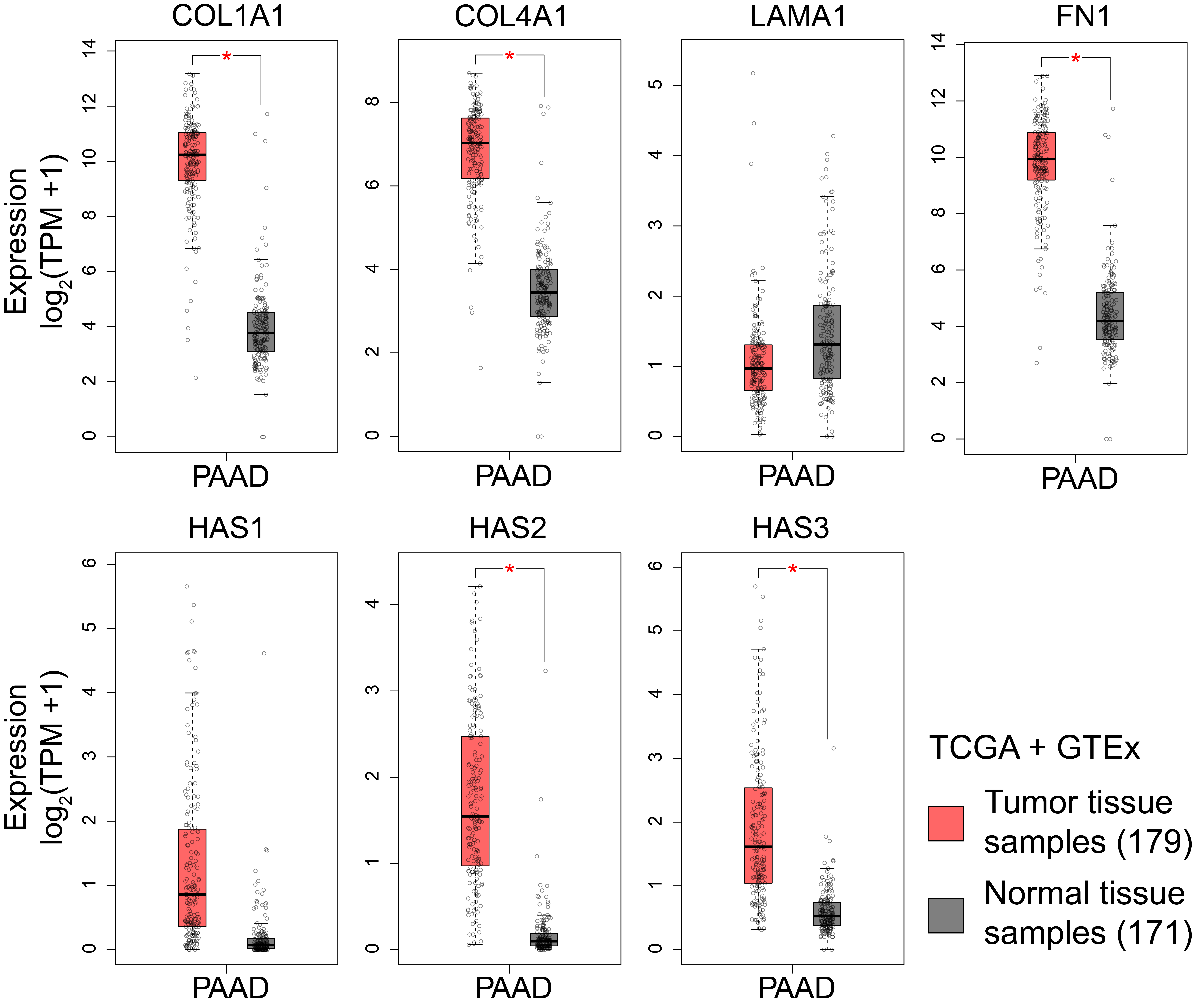


**Figure S1. Expression of selected key ECM components in pancreatic tumor and normal tissue.** Difference between expression values of selected genes represented by synthetic peptides in pancreatic tumor and adjacent normal tissues. Data extracted from The Cancer Genome Atlas (TCGA) and Genome-Tissue Expression project (GTEx). Data retrieved using GEPIA2 web-based analysis tool. Box and whiskers: boxes represent the 25th, 50th and 75th percentiles; lower and upper whiskers represent 5-95 percentiles, one-way ANOVA using disease state as variable, **p*<0.05.


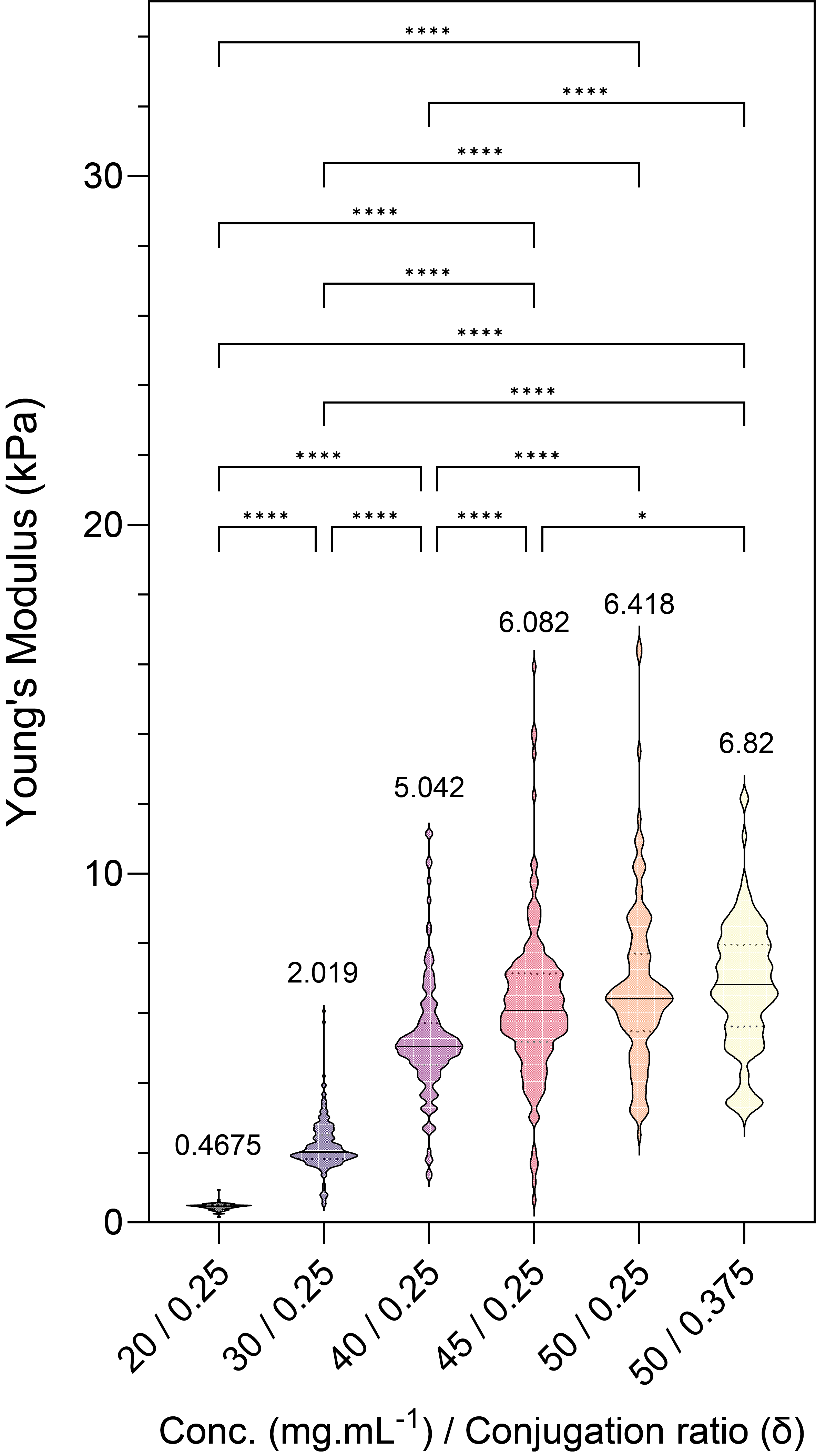


**Figure S2. Stiffness of synthetic hydrogels conjugated with peptides measured by AFM.** Young’s modulus of synthetic hydrogels 24h after incubation in DMEM with 10% (v/v) FBS and 1% Penicillin-Streptomycin to reach equilibrium swelling. Hydrogels with different solid contents and different conjugation ratios were subjected to indentation. Violin plots represent accumulated measurement points for 7×7 measurement regions of n=3-4 independent samples. Dashed lines are first and third quartiles, solid lines are median values, one-way ANOVA with Tukey’s post hoc test. **p* < 0.05, *****p* < 0.0001.


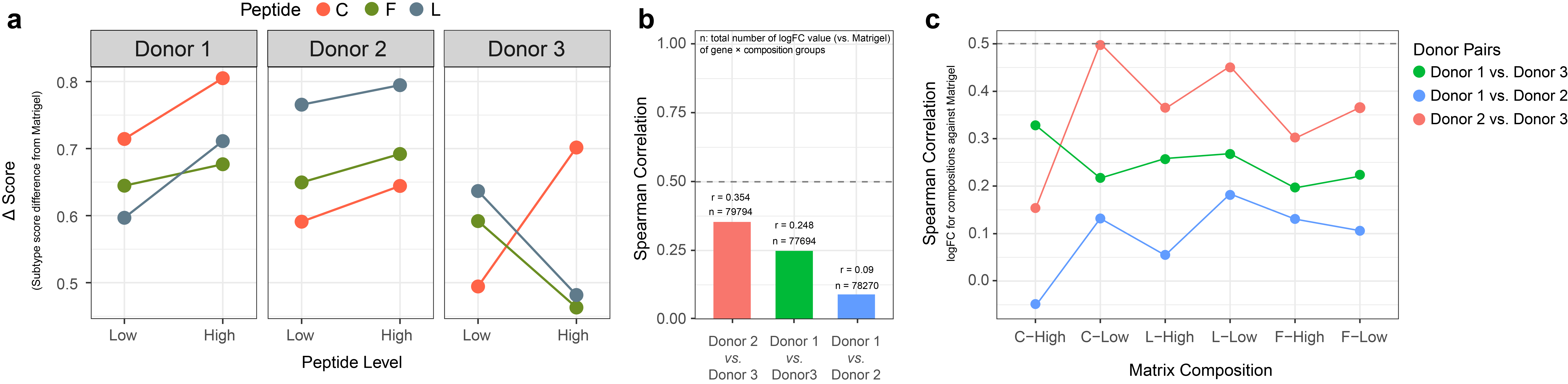


**Figure S3. Baseline-anchored basal-classical axis shifts and donor-dependent concordance of transcriptomic response signatures across matrix compositions.** **a.** Computed donor-wise subtype scores relative to Matrigel for each composition (Δ Score). The synthetic compositions are grouped for visualization into “High” and “Low” categories by the indicated label to aid readability. **b.** Spearman correlations of donor-specific transcriptomic response profiles across all (gene × composition) response points, where each response point was defined as composition vs. Matrigel within the donor. The bars show the resulting correlation coefficient for each donor pair. **c.** Data showing response concordance across both donor pairs and compositions. Only partial conservation of transcriptomic response program across patients under identical matrix conditions can be inferred.


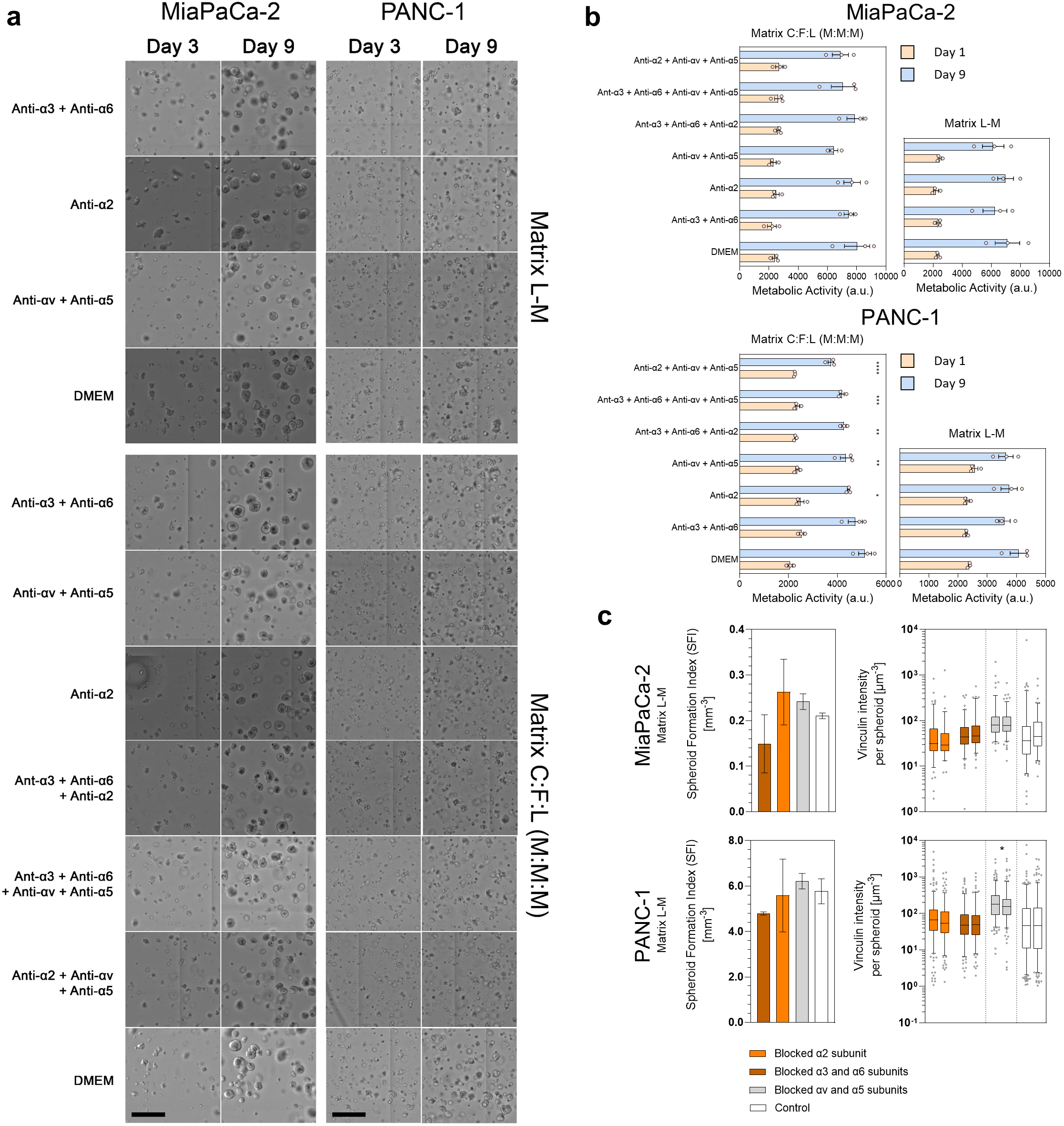


**Figure S4. Blocking integrin-mediated cell adhesion in synthetic matrices alters cytoskeletal organization and morphological features.** **a.** Time-lapse brightfield images show the formation of spheroids in mono- and multipeptide synthetic matrices, in response to blocking different integrin α subunits. Images correspond to 1 and 7 days after starting the treatment (Day 3 and Day 9 of culture). Faint straight boundaries visible in some tiles reflect the automated mosaic-processing workflow (stitching overlapping fields followed by illumination normalization) and are not the result of manual splicing or modification of the raw image data. Scale bars, 500 µm. **b.** Metabolic activity of encapsulated MiaPaCa-2 and PANC-1 cells in mono- and multipeptide synthetic matrices subjected to different anti-integrin α subunit treatments. (n = 3 independent samples, bars: mean ± SEM, two-way ANOVA for group and time comparisons with Dunnett’s post hoc test. Astriks denote significant differences between groups at the same time point. *p < 0.05, **p < 0.01, ***p < 0.001, ****p < 0.0001). **c.** Spheroid Formation Index and normalized expression of vinculin in L-only synthetic matrices. Blocking αv and α5 subunits resulted in increased average expression of vinculin compared with the control in both cell lines. (n = 2 independent experimental replicates; box and whiskers: boxes represent the 25th, 50th and 75th percentiles; lower and upper whiskers represent 5-95 percentiles, nested one-way ANOVA, *p<0.05; bars: mean ± SEM, one-way ANOVA, *p<0.05).


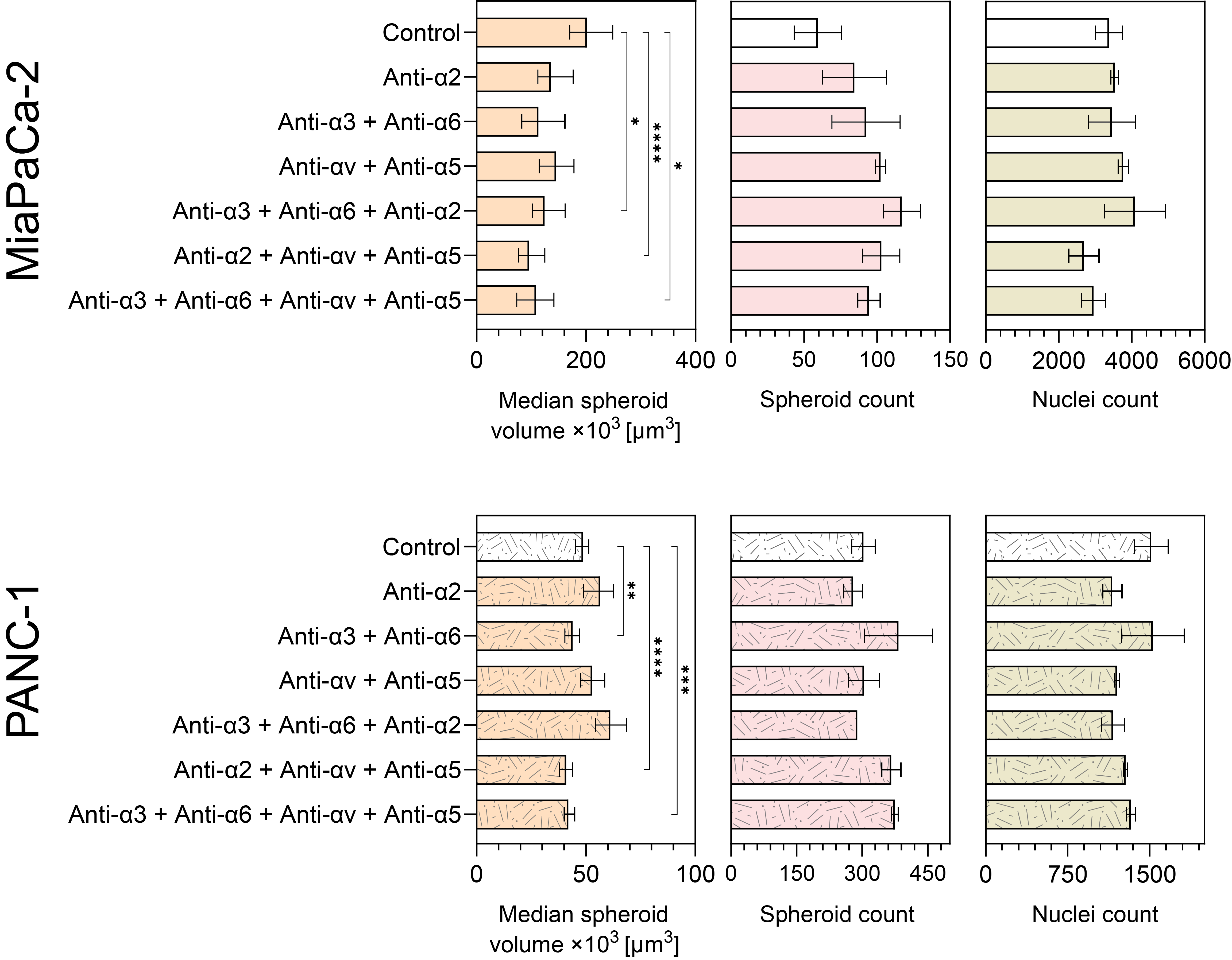


**Figure S5. Decomposed spheroid formation index parameters in multi-peptide cultures.** The spheroid formation index (SFI), a metric of fragmentation during culture, was defined using spheroid volume, the number of spheroids, and the number of cells in each culture. Respective values for each culture (MiaPaCa-2 and PANC-1) in response to integrin function-blocking treatments are displayed. Fragmentation or reduced compaction is operationally reflected by increased spheroid counts with reduced median spheroid volumes (often accompanied by reduced nuclei counts), which explains the direction of SFI changes. (n = 2 independent experimental replicates, bars (spheroid volume): median with 95% CI, one-way Welch ANOVA, *p<0.05, **p<0.01, ***p<0.001, ****p<0.0001, bars (spheroid and nuclei counts): mean ± SEM).


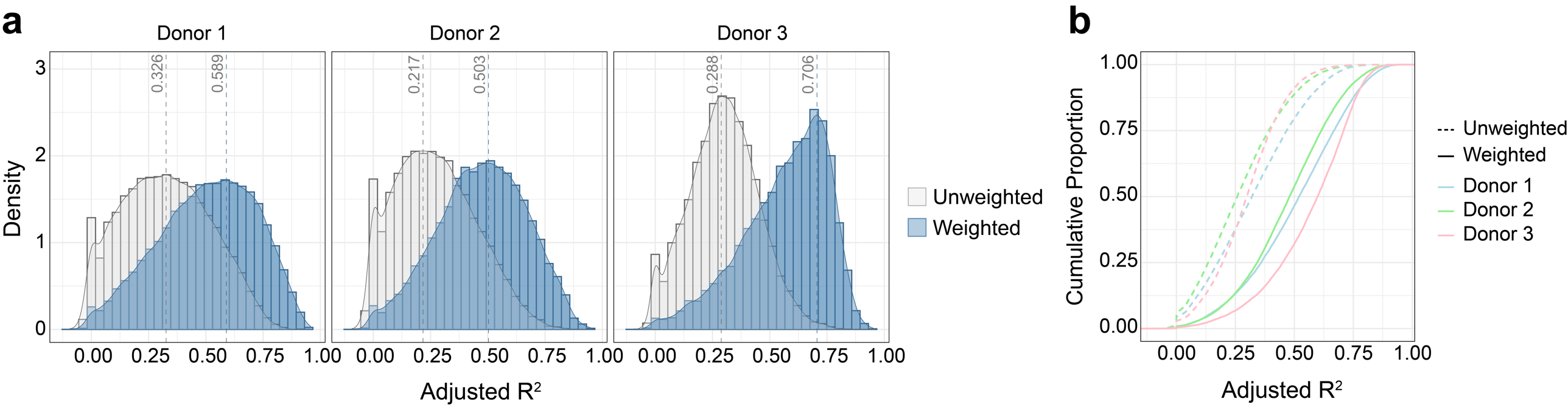


**Figure S6. Weighted regression in DoE modeling improved the models’ accuracy. a,** Density plot showing the distribution of adjusted-R^2^ values of fitted models across the whole transcriptome, using two regression approaches. Weighted regression was based on group-weighting to compensate for sample-level noise due to technical variability during sequencing. **b,** Empirical Cumulative Distribution Function (ECDF) plot showing the cumulative proportions of adjusted-R^2^ values in two regression methods.


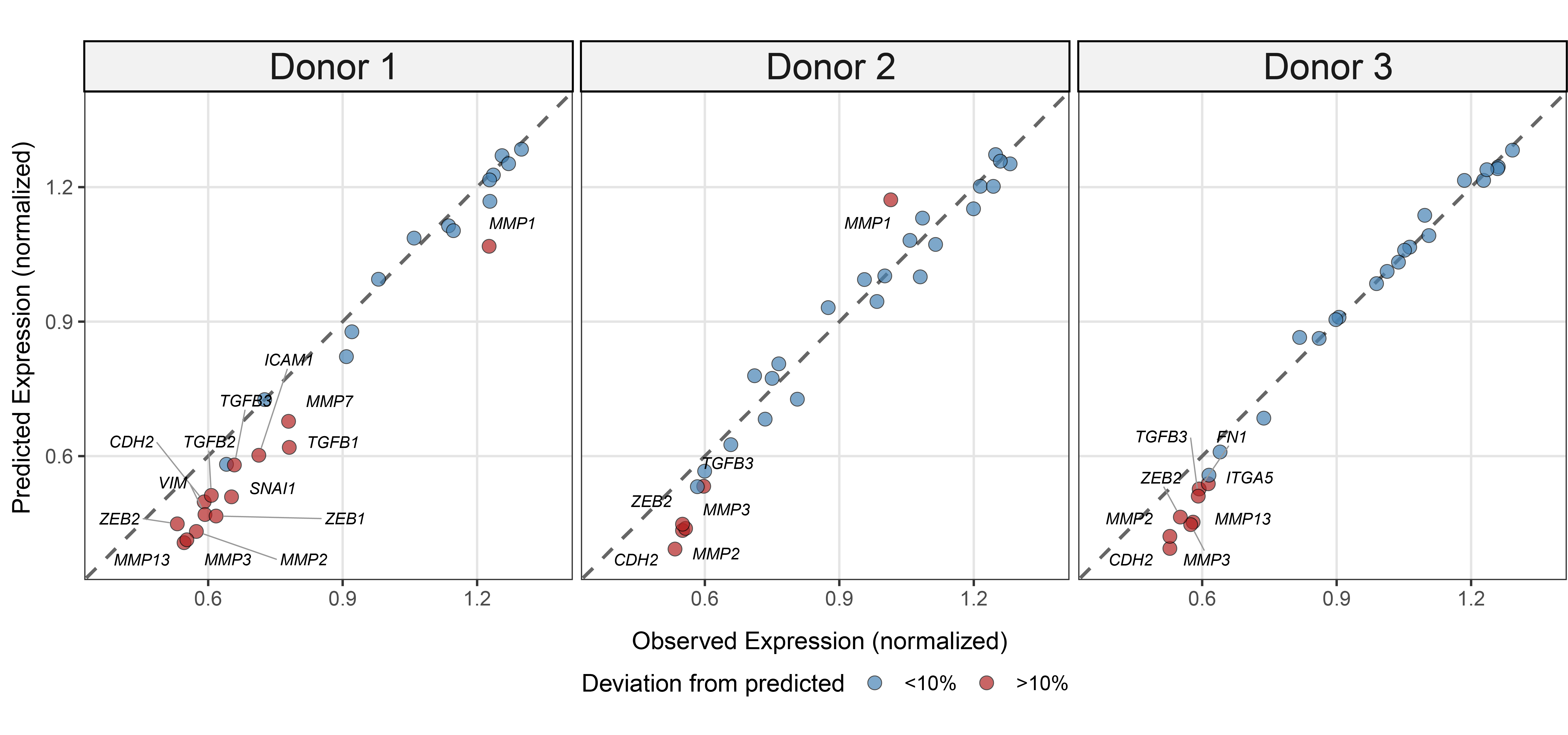


**Figure S7. Agreement between model predictions and observed EMT gene expression in EMT Designer matrices.** Scatter plots show, for each donor, the observed expression values (technical replicates averaged) versus the corresponding values predicted by the donor-specific response-surface models for the EMT objective gene set used in the multi-objective optimization. The dashed line indicates the identity line (perfect agreement). Points are colored based on whether each gene falls within a predefined relative deviation threshold of the prediction (10%), and genes exceeding the threshold are annotated for visual reference.

**
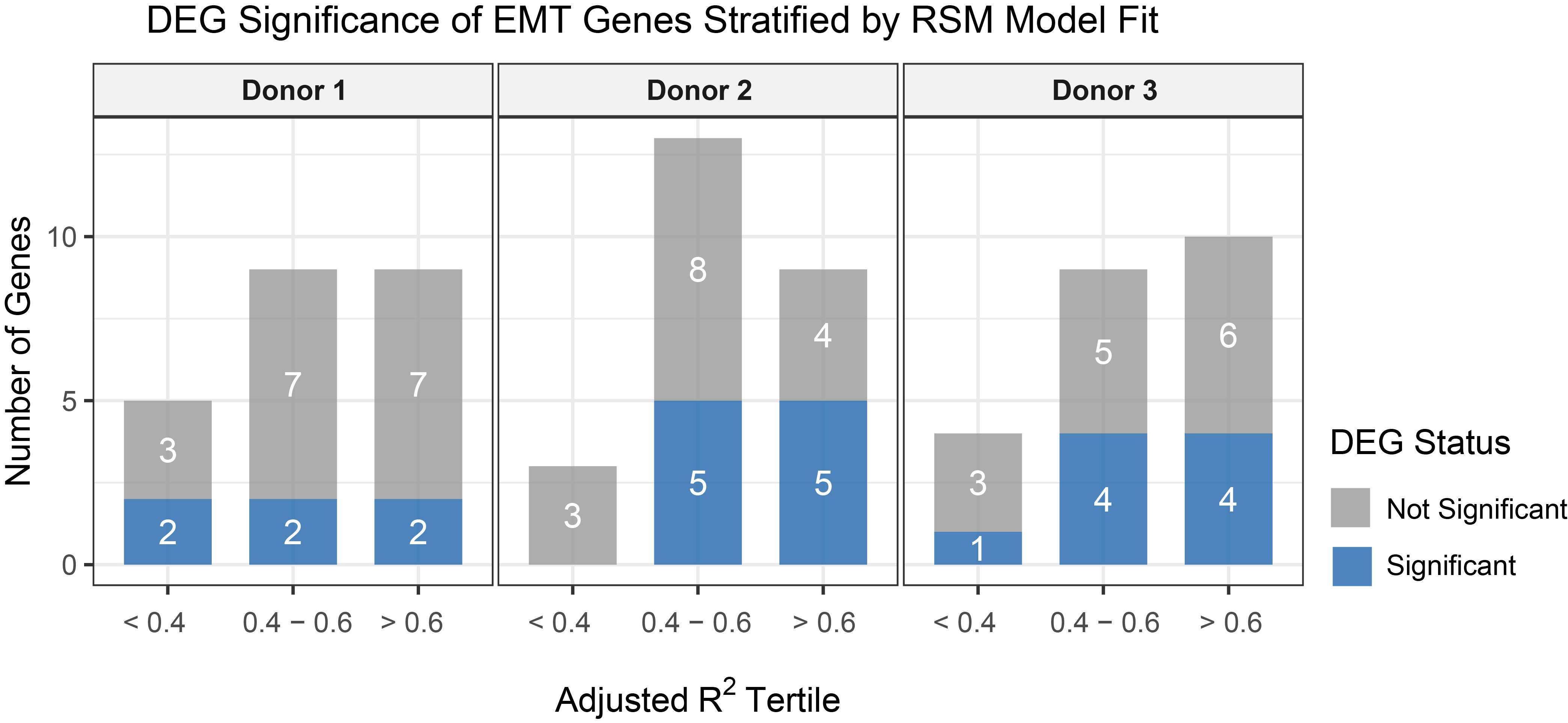
**

**Figure S8. Differential expression of EMT objective genes in EMT Designer stratified by DoE model reliability.** The number of DEGs across different tertiles of adjusted R^2^ values indicates donor-dependent, non-monotonic changes in response to the coordinated optimization approach. The relationship follows the network-level framing of the optimization. “EMT Genes” refer to the 29 genes used as objectives in the multiobjective optimization implementation.

**
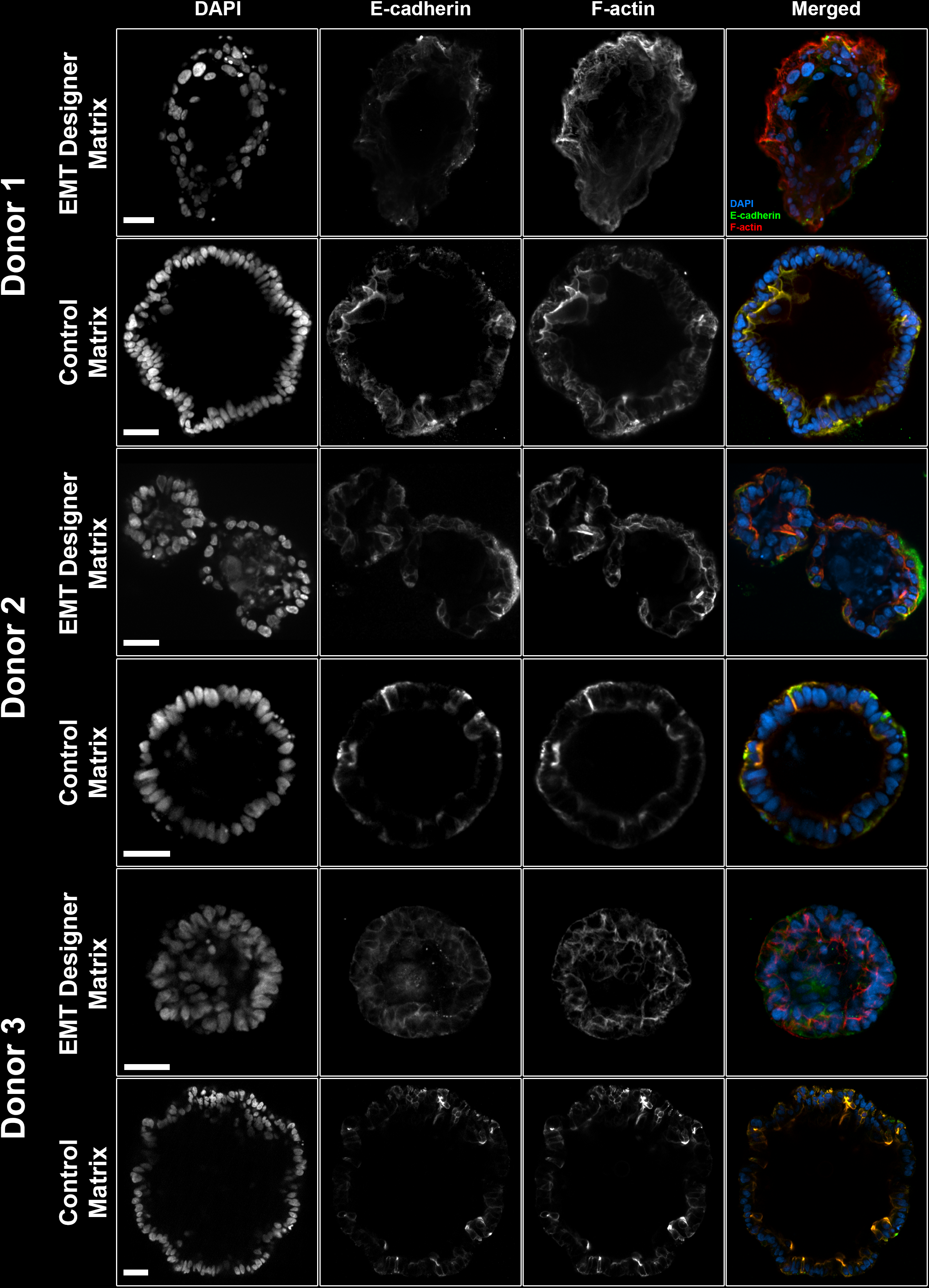
**

**Figure S9. Immunofluorescence imaging shows epithelial junction and cytoskeletal remodeling in organoids cultured in EMT Designer and control matrices.** Representative fluorescence images show, across donors, organoids cultured in the EMT Designer matrix frequently exhibit reduced continuity or redistribution of E-cadherin, together with altered F-actin architecture and reduced spatial coupling of these markers relative to controls, consistent with partial EMT-like junctional/cytoskeletal remodeling rather than a binary epithelial-to-mesenchymal conversion. The phenotype was heterogeneous across spheroids; images show representative examples. Scale bars, 30 µm.


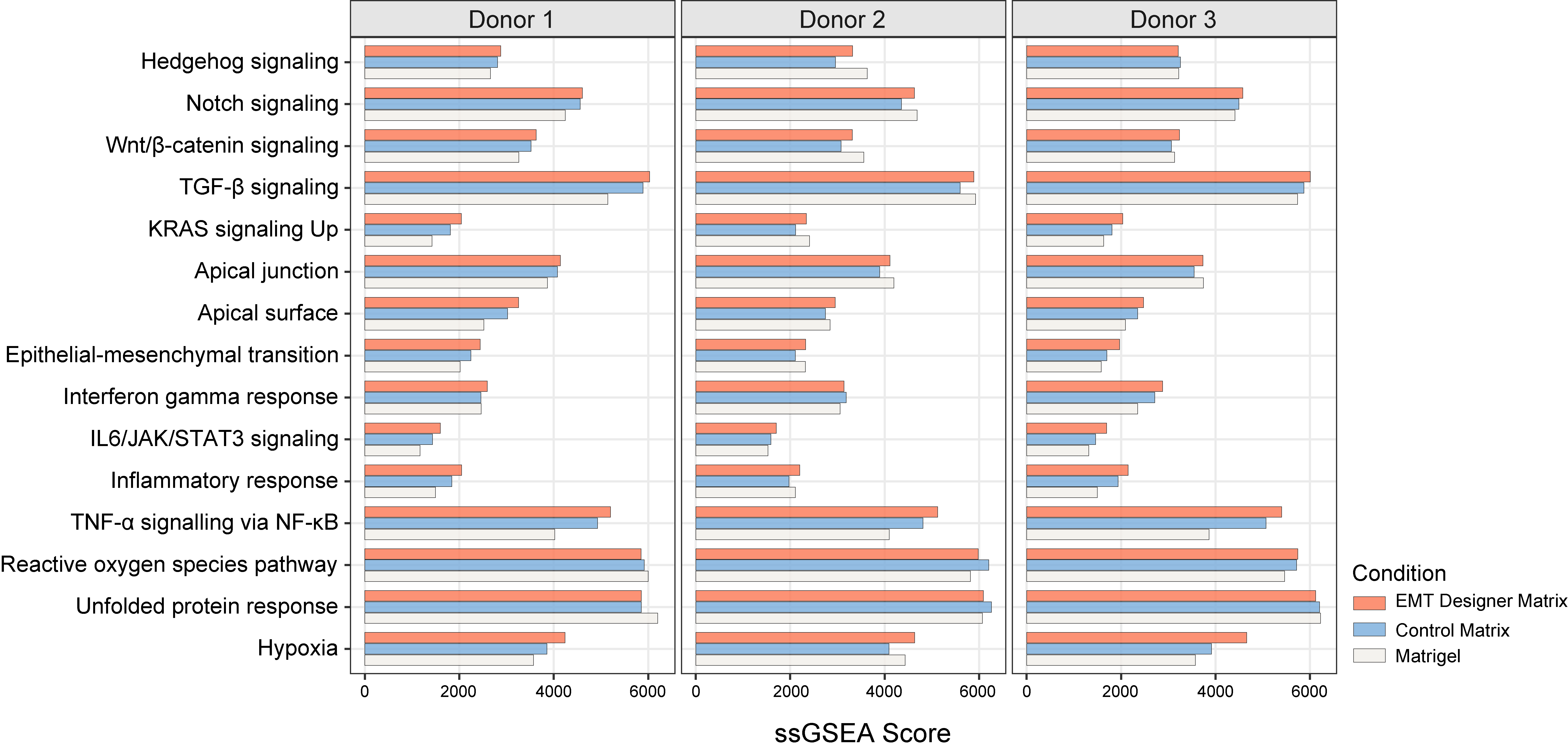


**Figure S10. ssGSEA scores for selected Hallmark gene sets from organoids cultured in EMT Designer and control matrices, as well as Matrigel as the standard organoid culture ECM.** The analysis contextualizes ECM-dependent transcriptional states across defined synthetic and natural ECM environments. EMT-associated and related signaling programs exhibit donor-dependent shifts across matrix conditions. Values represent average scores of three technical replicates.

**Supporting Table S1.** **Peptide combinations according to the central composite design (CCD) and the composition of validation experiments.** C, F, and L levels varied between the upper and lower limits of the design space, including six axial points. The content of B was kept constant in all the experimental runs. A validation set, including five randomly picked combinations of the C, F, and L, was used to evaluate modelling performance. Experimental runs were randomized to prevent systemic bias.

| **C [mM]** | **F [mM]** | **L [mM]** | **B [mM]** |  |
| --- | --- | --- | --- | --- |
| 0.75 | 0.15 | 0.15 | 0.2625 | **Experimental Runs** |
| 1.5 | 0.15 | 0.15 | 0.2625 |  |
| 0.75 | 0.375 | 0.15 | 0.2625 |  |
| 0.75 | 0.15 | 0.375 | 0.2625 |  |
| 1.5 | 0.375 | 0.15 | 0.2625 |  |
| 1.5 | 0.15 | 0.375 | 0.2625 |  |
| 0.75 | 0.375 | 0.375 | 0.2625 |  |
| 1.5 | 0.375 | 0.375 | 0.2625 |  |
| 1.125 | 0.2625 | 0.2625 | 0.2625 |  |
| 1.125 | 0.2625 | 0.2625 | 0.2625 |  |
| 1.125 | 0.2625 | 0.2625 | 0.2625 |  |
| 1.125 | 0.2625 | 0.2625 | 0.2625 |  |
| 1.125 | 0.2625 | 0.2625 | 0.2625 |  |
| 1.125 | 0.2625 | 0.2625 | 0.2625 |  |
| 1.125 | 0.2625 | 0.2625 | 0.2625 |  |
| 1.125 | 0.2625 | 0.2625 | 0.2625 |  |
| 1.125 | 0.2625 | 0.2625 | 0.2625 |  |
| 1.125 | 0.2625 | 0.074846 | 0.2625 |  |
| 1.125 | 0.2625 | 0.450154 | 0.2625 |  |
| 1.125 | 0.074846 | 0.2625 | 0.2625 |  |
| 1.125 | 0.450154 | 0.2625 | 0.2625 |  |
| 0.499488 | 0.2625 | 0.2625 | 0.2625 |  |
| 1.750512 | 0.2625 | 0.2625 | 0.2625 |  |
| 1.0 | 0.18 | 0.32 | 0.2625 | **Confirmation** |
| 0.8 | 0.35 | 0.18 | 0.2625 |  |
| 1.5 | 0.2 | 0.2625 | 0.2625 |  |
| 0.75 | 0.32 | 0.35 | 0.2625 |  |
| 1.3 | 0.3 | 0.3 | 0.2625 |  |

**Supporting Table S2.** **Prediction accuracy of the EMT Designer Matrices in inducing EMT for different donors.** The expression of the EMT-associated genes from the optimized set for organoids cultured in EMT Designer Matrices from different donors shows high agreement with the predicted values.

| **Donor** | **R^*^** | | **RMSE^**^** | | **Mean sMAPE^***^** | **Fraction sMAPE^****^** | | | |
| --- | --- | --- | --- | --- | --- | --- | --- | --- | --- |
|  | **Mean** | **SD** | **Mean** | **SD** |  | **< 0.1** | **< 0.2** | **< 0.3** |  |
| **Donor 1** | 0.990 | 1.11E-16 | 0.0923 | 7.93E-04 | 0.1190 | 0.4760 | 0.7500 | 0.9880 |  |
| **Donor 2** | 0.977 | 8.16E-04 | 0.0691 | 7.12E-04 | 0.0728 | 0.7263 | 0.8570 | 0.9760 |  |
| **Donor 3** | 0.994 | 1.11E-16 | 0.0602 | 8.04E-04 | 0.0810 | 0.7140 | 0.8450 | 1.0000 |  |

* Pearson’s correlation coefficient

** Root Mean Squared Error

*** Symmetric Mean Absolute Percentage Error

**** Fraction of genes below the threshold of sMAPE

**Supporting Table S3.** **Curated list of genes associated with EMT in pancreatic cancer.** The marked direction of change for each gene was further considered during the multiobjective optimization step.

| **Gene** | **Marked direction of change** |
| --- | --- |
| SNAI1 | Upregulated |
| SNAI2 | Upregulated |
| ZEB1 | Upregulated |
| ZEB2 | Upregulated |
| VIM | Upregulated |
| NANOG | Upregulated |
| CDH2 | Upregulated |
| FN1 | Upregulated |
| MMP1 | Upregulated |
| MMP2 | Upregulated |
| MMP3 | Upregulated |
| MMP7 | Upregulated |
| MMP13 | Upregulated |
| MMP14 | Upregulated |
| ITGAV | Upregulated |
| LAMC2 | Upregulated |
| NEDD9 | Upregulated |
| HSPG2 | Upregulated |
| ITGA5 | Upregulated |
| ICAM1 | Upregulated |
| TGFB1 | Upregulated |
| TGFB2 | Upregulated |
| TGFB3 | Upregulated |
| CDH1 | Downregulated |
| OCLN | Downregulated |
| CLDN1 | Downregulated |
| DSP | Downregulated |
| JUP | Downregulated |
| EPCAM | Downregulated |
